# Optimal subthalamic stimulation sites and related networks for freezing of gait in Parkinson’s disease

**DOI:** 10.1093/braincomms/fcad238

**Published:** 2023-09-04

**Authors:** Houyou Fan, Zijian Guo, Yin Jiang, Tao Xue, Zixiao Yin, Hutao Xie, Yu Diao, Tianqi Hu, Baotian Zhao, Delong Wu, Qi An, Yichen Xu, Yuan Gao, Yutong Bai, Jianguo Zhang

**Affiliations:** Department of Neurosurgery, Beijing Tiantan Hospital, Capital Medical University, 100070 Beijing, China; Department of Functional Neurosurgery, Beijing Neurosurgical Institute, Capital Medical University, 100070 Beijing, China; Department of Neurosurgery, Beijing Tiantan Hospital, Capital Medical University, 100070 Beijing, China; School of Biomedical Engineering, Capital Medical University, 100069 Beijing, China; Department of Functional Neurosurgery, Beijing Neurosurgical Institute, Capital Medical University, 100070 Beijing, China; Beijing Key Laboratory of Neurostimulation, 100070 Beijing, China; Department of Neurosurgery, Beijing Tiantan Hospital, Capital Medical University, 100070 Beijing, China; Department of Functional Neurosurgery, Beijing Neurosurgical Institute, Capital Medical University, 100070 Beijing, China; Department of Neurosurgery, Beijing Tiantan Hospital, Capital Medical University, 100070 Beijing, China; Department of Functional Neurosurgery, Beijing Neurosurgical Institute, Capital Medical University, 100070 Beijing, China; Department of Neurosurgery, Beijing Tiantan Hospital, Capital Medical University, 100070 Beijing, China; Department of Functional Neurosurgery, Beijing Neurosurgical Institute, Capital Medical University, 100070 Beijing, China; Department of Neurosurgery, Beijing Tiantan Hospital, Capital Medical University, 100070 Beijing, China; Department of Functional Neurosurgery, Beijing Neurosurgical Institute, Capital Medical University, 100070 Beijing, China; Department of Neurosurgery, Beijing Tiantan Hospital, Capital Medical University, 100070 Beijing, China; Department of Functional Neurosurgery, Beijing Neurosurgical Institute, Capital Medical University, 100070 Beijing, China; Department of Neurosurgery, Beijing Tiantan Hospital, Capital Medical University, 100070 Beijing, China; Department of Functional Neurosurgery, Beijing Neurosurgical Institute, Capital Medical University, 100070 Beijing, China; Beijing Key Laboratory of Neurostimulation, 100070 Beijing, China; Department of Neurosurgery, Beijing Tiantan Hospital, Capital Medical University, 100070 Beijing, China; Department of Functional Neurosurgery, Beijing Neurosurgical Institute, Capital Medical University, 100070 Beijing, China; Department of Neurosurgery, Beijing Tiantan Hospital, Capital Medical University, 100070 Beijing, China; Department of Functional Neurosurgery, Beijing Neurosurgical Institute, Capital Medical University, 100070 Beijing, China; Department of Neurosurgery, Beijing Tiantan Hospital, Capital Medical University, 100070 Beijing, China; Department of Functional Neurosurgery, Beijing Neurosurgical Institute, Capital Medical University, 100070 Beijing, China; Department of Functional Neurosurgery, Beijing Neurosurgical Institute, Capital Medical University, 100070 Beijing, China; Beijing Key Laboratory of Neurostimulation, 100070 Beijing, China; Department of Neurosurgery, Beijing Tiantan Hospital, Capital Medical University, 100070 Beijing, China; Department of Functional Neurosurgery, Beijing Neurosurgical Institute, Capital Medical University, 100070 Beijing, China; Department of Neurosurgery, Beijing Tiantan Hospital, Capital Medical University, 100070 Beijing, China; Department of Functional Neurosurgery, Beijing Neurosurgical Institute, Capital Medical University, 100070 Beijing, China; Beijing Key Laboratory of Neurostimulation, 100070 Beijing, China

**Keywords:** Parkinson’s disease, freezing of gait, subthalamic deep brain stimulation, optimal simulation site, connectivity network

## Abstract

Freezing of gait is a common and debilitating symptom in Parkinson’s disease. Although high-frequency subthalamic deep brain stimulation is an effective treatment for Parkinson’s disease, post-operative freezing of gait severity has been reported to alleviate, deteriorate or remain constant. We conducted this study to explore the optimal stimulation sites and related connectivity networks for high-frequency subthalamic deep brain stimulation treating freezing of gait in Parkinson’s disease. A total of 76 Parkinson’s disease patients with freezing of gait who underwent bilateral high-frequency subthalamic stimulation were retrospectively included. The volumes of tissue activated were estimated based on individual electrode reconstruction. The optimal and sour stimulation sites were calculated at coordinate/voxel/mapping level and mapped to anatomical space based on patient-specific images and stimulation settings. The structural and functional predictive connectivity networks for the change of the post-operative Freezing of Gait-Questionnaire were also identified based on normative connectomes derived from the Parkinson’s Progression Marker Initiative database. Leave-one-out cross-validation model validated the above results, and the model remained significant after including covariates. The dorsolateral two-thirds of the subthalamic nucleus was identified as the optimal stimulation site, while the ventrocentral portion of the right subthalamic nucleus and internal capsule surrounding the left central subthalamic nucleus were considered as the sour stimulation sites. Modulation of the fibre tracts connecting to the supplementary motor area, pre-supplementary motor area and pedunculopontine nucleus accounted for the alleviation of freezing of gait, whereas tracts connecting to medial and ventrolateral prefrontal cortices contributed to the deterioration of freezing of gait. The optimal/sour stimulation sites and structural/functional predictive connectivity networks for high-frequency subthalamic deep brain stimulation treating freezing of gait are identified and validated through sizable Parkinson’s disease patients in this study. With the growing understanding of stimulation sites and related networks, individualized deep brain stimulation treatment with directional leads will become an optimal choice for Parkinson’s disease patients with freezing of gait in the future.

## Graphical Abstract

**Graphical abstract:**
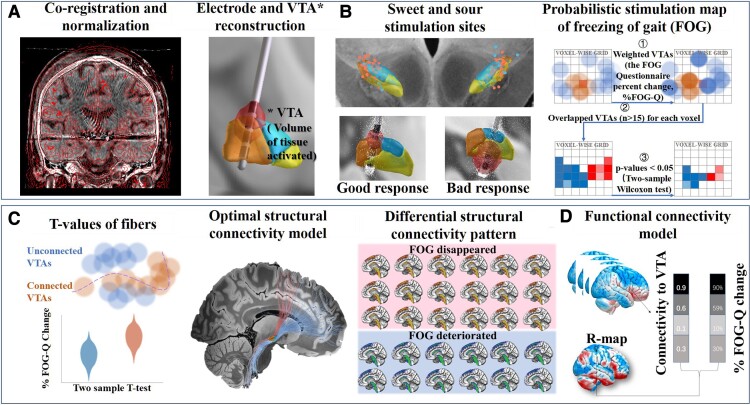

## Teaser

Fan *et al*. report a *post hoc* analysis of high-frequency subthalamic deep brain stimulation outcome on freezing of gait in relation to stimulation sites and related brain networks, and the dorsolateral two-thirds of the subthalamic nucleus is recommended for freezing of gait.

## Introduction

Freezing of gait (FOG) is a common and debilitating symptom in Parkinson’s disease that is characterized by sudden and transient episodes of walking or turning arrest.^1^ The incidence of FOG significantly increases with the progression of Parkinson’s disease, leading to a higher risk of falls. The prevalence of FOG exceeds 50% in advanced Parkinson’s disease patients and exceeds 90% in patients at Hoehn and Yahr (H–Y) stage 4.^2^ After focusing on a one-axis mechanism underlying FOG in isolation, it has recently been recommended to take a holistic view of the heterogeneous FOG symptoms, in which the abnormalities of cognitive, motor and affective networks might all have significant roles in the pathophysiology of FOG.^3^

Subthalamic deep brain stimulation (STN-DBS) is an effective treatment for advanced Parkinson’s disease because STN-DBS effectively controls motor symptoms such as rigidity and tremor and reduces the dosage of levodopa intake.^4^ There is an interesting and contradictory phenomenon whereby post-operative FOG severity has been reported to alleviate, deteriorate, or remain constant among different Parkinson’s disease patients.^5,6^ Though the FOG symptom of many patients is dramatically alleviated after STN-DBS, one-third of patients have symptom exacerbation.^4,5^ Some patients even develop new-onset FOG after STN-DBS surgery. The outcome variability has been reported to be mainly determined by variations in electrode location and incorrect stimulation settings.^7–9^ Some recent small-sample studies reported a significant association between FOG changes and the recruitment of sensorimotor and prefrontal dorsolateral cortico-subthalamic fibres, as well as the hyper-direct pathway connecting to supplementary area (SMA).^10,11^ We recently proposed a bandwidth model supporting that STN-DBS improves FOG by both reducing motor impairments and enhancing the overall cortical information-processing capabilities.^12^ Except for cortico-subthalamic connectivity, it has been suggested that STN-DBS improves Parkinsonian gait by modulating pedunculopontine nucleus/mesencephalic locomotor region (PPN/MLR) activity.^13^ However, the mechanisms underlying the paradoxical outcome of FOG-DBS treatment are still a matter of debate, requiring further studies from larger samples and multiple perspectives.

Given these studies, we speculated that STN-DBS alleviated or deteriorated FOG by stimulating different STN subregions and engaging in different brain networks. We used individual image data, such as pre-operative MRI and post-operative CT scans, the volume of tissue activated (VTA) model combining stimulation location and parameter settings and normative connectomes derived from diffusion and functional MRI to identify the optimal stimulation sites and related connectivity networks of STN-DBS on FOG in Parkinson’s disease.

## Materials and methods

### Patients and clinical characteristics

#### Patient screening and clinical data

Parkinson’s disease patients who underwent bilateral STN-DBS at Beijing Tiantan Hospital from June 2018 to June 2021 were screened retrospectively. All patients were strictly included only after meeting the following inclusion criteria: (i) pre-operative FOG-Questionnaire (FOG-Q) item 3 ≥1; (ii) assessment videos are available and at least one freezing episode was observed in the video; (iii) available parameter settings and a stimulation frequency ≥90 Hz; and (iv) available MRI and post-surgical CT. Patients were excluded for the following reasons: (i) other brain diseases leading to FOG; (ii) lower limb disability; (iii) history of stereotactic-lesioning surgery or DBS of other stimulation targets; (iv) severe surgical adverse effects and/or stimulation-related complications; and (v) ON-state FOG.

The surgical procedure was described in Supplementary Methods. Every patient was at his/her best therapeutic stimulation setting during the follow-up assessment. The mechanisms underlying STN-DBS with different frequencies are still under debate.^9^ The local and global effects on brain changes were also regarded to be different.^14^ Therefore, we only collected patients with high-frequency STN-DBS to exclude the analysis bias of frequency difference. Sex, disease duration, age at onset/surgery, H–Y stage, Movement Disorder Society Unified Parkinson’s Disease Rating Scale (MDS-UPDRS), Levodopa Equivalent Daily Dose (LEDD), Berg Balance Scale, Mini-mental State Exam (MMSE), Montreal Cognitive Assessment (MoCA), 14-item Hamilton Anxiety Scale (HAM-A), 24-item Hamilton Depression Scale (HAM-D), therapeutic stimulation settings and other important clinical data were collected pre- operatively and post-operatively. This study followed the Declaration of Helsinki and was approved by the Institutional Review Board of Beijing Tiantan Hospital. All subjects provided written informed consent.

#### Freezing of gait assessment and grouping

The FOG-Q is a questionnaire that assesses the normal and worst FOG symptom in the most recent 1 month, and all patients were assessed at medication off state to help patients better recall and answer the questions.^15^ The FOG-Q percent change was calculated as follows: %FOG-Q change = [(pre-operative FOG-Q score–post-operative FOG-Q score)/pre-operative FOG-Q] × 100. An FOG-Q reduction was considered a positive FOG-Q change because FOG represented the alleviation of FOG severity from baseline to follow-up was calculated as the primary outcome. The effect of disease progression was excluded by calculating whether %FOG-Q was related with follow-up duration. For subgroup comparisons, patients were delivered into three subgroups, including alleviation, minor change and deterioration. The cut-off values of %FOG-Q change were 0 and 30% because the change rate below 0 is generally deemed as a deterioration of symptoms and >30% is considered good improvement.^16^ Patients in the alleviation group whose FOG-Q Item 3 (‘frequency of freezing episodes’) score changed to 0 after STN-DBS were further classified into the FOG-disappearance group for validation analysis.

#### Imaging data collection

All patients underwent pre-operative MRI and post-operative CT imaging scans. A 3-Tesla MRI scanner (Philips Medical Systems, Best, The Netherlands) was used to acquire T1-weighted scans [slice thickness, 1 mm; repetition time (TR), 6.9 ms; echo time (TE), 3.1 ms]. The slice thickness of the post-operative CT was 0.625 mm. For the Parkinson’s Progression Marker Initiative (PPMI) database (www.ppmi-info.orgz) that was used to generate normative connectomes, 90 Parkinson’s disease patients underwent diffusion-weighted scans (TR/TE = 900/88 ms; 2 mm^3^ resolution; 72 slices; 64 gradient directions; and b = 1000 s/mm^2^) and resting fMRI scans (TR/TE = 2400/25 ms; flip angle = 80°; field of view = 240 × 240 mm; matrix = 68 × 68; 40 slices; slice thickness = 3.3 mm; voxel size = 3.25 × 3.25 × 3.25 mm^3^; 212 volumes).^17^ The processes of converting these original imaging data to available normative connectomes are described in detail in the Supplementary Methods sections according to Horn *et al*.^18^

### Image pre-processing and computational analysis

#### Electrode localization

**Figure 1:**
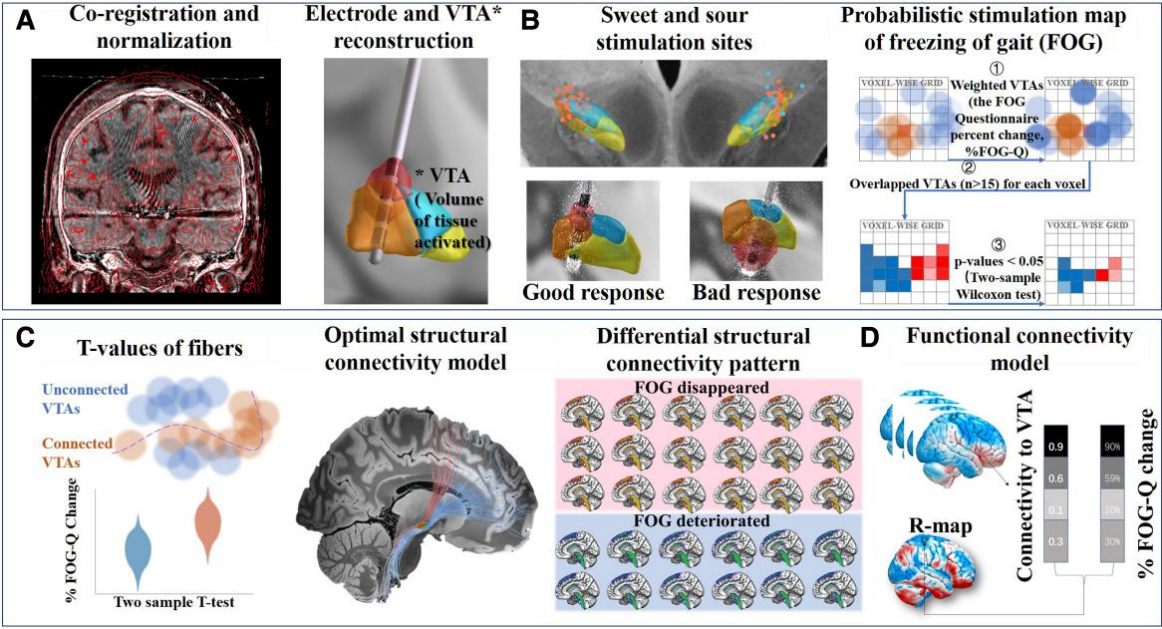
Illustration of the methodology. **(A)** Electrodes (132 sides) were reconstructed by the co-registration and alignment of pre-operative MRI (T1 and T2) and post-operative CT. The activated contacts and stimulation parameters were used to estimate bilateral VTAs. **(B)** The MNI coordinates of activated contacts were all extracted, and the correlation with FOG-Q change was estimated. Then, the relationships between the intersections of VTAs with STN/subregions and FOG-Q changes were calculated. The PSM was also identified by combining voxels of VTAs and FOG-Q change. **(C)** The stimulated fibre tracts by VTAs of each patient were all identified based on the PPMI dataset. The relationship between individual connection status and the patient-specific FOG-Q outcome was calculated using a linear mixed model; the positive (red) and negative (blue) fibres were identified by effect sizes. FOG-disappearance and FOG-deterioration patients were selected to process the sensitive analysis to extract the different fibres. **(D)** The functional connectivity pattern per patient was identified based on the PPMI dataset. Then, the R-map was generated by correlating the individual functional connectivity pattern with the FOG-Q changes at the voxel-wise level.

Figure 1 illustrates the methodology used in this study. Briefly, a pre-operative MRI and post-operative CT scans were linearly co-registered and non-linearly normalized into the Montreal Neurological Institute (MNI) ICBM 2009b NLIN ASYM template space using advanced normalization tools.^19^ Then, the brain shift correction was performed, and all of the above outcomes were inspected by experienced neurosurgeons (HF and YB, respectively).^20^ The Precise and Convenient Electrode Reconstruction (PaCER) and refined TRAC/CORE methods were applied to pre-construct the electrodes, which were manually inspected and subsequently refined by HF and YB.^21^ The DBS Intrinsic Template AtLas (DISTAL) minimal atlas was used to visualize the STN, and this atlas was very accurate for localizing the basal ganglia structure because the atlas was designed for surgical localization.^22^ To graphically illustrate the electrode locations, 2D slices were plotted using the 7-T 100-μm *ex vivo* human brain MRI template as a background image. The reconstruction processes were conducted in the Lead-DBS toolbox (version 2.5.3).^23^

#### Activated contact coordinate extraction and volume of tissue activated estimation

Three-dimensional coordinates of the activated contacts of the DBS electrodes in the MNI space were then extracted from the reconstructed electrode models. The VTA was estimated in native space based on the finite element method.^23^ Patient-specific stimulation parameters, especially activated contacts, pulse width, frequency and amplitudes, were used to calculate VTAs using the SimBio/FieldTrip pipeline.^24^ A volume conductor model was constructed based on a four-compartment mesh that includes grey matter (*σ* = 0.33 S/m), white matter (*σ* = 0.14 S/m), electrode contacts and insulated sections (Fig. 1A). Binary VTAs were generated by thresholding the gradient vector magnitude distribution at 0.2 V/mm.

#### Sweet and sour stimulation sites

⧫ Coordinates-level analysis: Spearman’s coefficients of activated contact coordinates with the %FOG-Q changes were calculated. It is essential to emphasize that relative coordinates do not accurately reflect the exact position of the contacts (for instance, if contact A was more ventral compared to contact B, it did not entail that contact A was in the ventral part of the STN and anatomy must be taken into consideration). Therefore, comparisons between subgroups were further conducted (Fig. 1B left).
⧫ VTA-level analysis: STN was divided into three subregions according to the DISTAL minimal atlas (sensorimotor, associative and limbic STN).^22^ The overlap volumes between the VTAs and STN/subregions were extracted to calculate the correlation with the %FOG-Q changes (Fig. 1B left).
⧫ Mapping-level analysis: A VTA-based probabilistic stimulation map (PSM) was also calculated to identify voxels that produced above-average (‘sweet spots’) and below-average (‘sour spots’) clinical changes when stimulated.^25^ Each patient’s VTAs were weighted by their %FOG-Q change. Subsequently, the average value of %FOG-Q changes was obtained for each voxel. Available voxels should be covered by a minimum of 20% of the included patients’ VTAs to ensure the validity of voxel-wise statistics. For each voxel, two-tailed Wilcoxon signed rank tests were applied to compare clinical outcomes between connected and disconnected VTAs to generate *P*-values (Fig. 1B right). Finally, we created the PSM (also named as the ‘significant mean effect images’) by only including voxels, which showed significant results (*P* < 0.05). The probabilistic mapping scores were calculated by the sum of all clinically weighted voxels in the overlap areas between per patient’s VTAs and the PSM. VTAs with larger overlap with the ‘sweet spots’ and less overlap with the ‘sour spots’ would be associated with better FOG improvement. Leave-one-out cross-validation (LOOCV) was applied to validate the PSM model. To control for the influence of sex, disease duration, pre-op MoCA, pre-op HAM-A/HAM-D, LEDD reduction and UPDRS-III percent change, those variables were included as covariates. Then, the coordinates of the centroid and peek-point of sweet and sour spots in PSM were calculated and extracted.

#### Structural connectivity analysis

The structural connectivity network between VTAs and all other brain areas was calculated using the normative connectome base on the PPMI dataset that has been proven to effectively explore the effects of DBS in previous studies.^18,23,26^ The DBS fibre-filtering method was applied to identify white matter fibre tracts overlapped by the VTAs. In each subject, a total of 20 000 fibres were sampled using a generalized q-sampling approach implemented in DSI Studio.^18^ Fibres that were connected to a minimum of 20% of the included patients’ VTAs were used for further analysis. For each fibre, a ‘Fibre-T-score’ was assigned by calculating two-sample *t*-tests to compare clinical outcomes between connected and disconnected VTAs (Fig. 1C left). These T-scores were not intended to calculate significant results but instead to identify a predictive model (Fig. 1C middle). Positive T-scores stand for preferentially stimulated fibre tracts in well-responded patients and negative T-scores for the preferentially stimulated ones in poor-responded patients. LOOCV was used for validation. Sex, disease duration, pre-op MoCA, pre-op HAM-A/HAM-D, LEDD reduction and UPDRS-III percent change were included as covariates to exclude confounders. Subgroups of FOG-disappearance and FOG-deterioration patients were included for further confirmatory analysis. To determine the differences between these two groups, a patient-specific structural connectivity pattern was used for comparative analysis between groups by independent samples *t*-test using DPABI (version 6.1) (Fig. 1C right).

#### Functional connectivity analysis

Analyses of structural connectivity are only capable of identifying monosynaptic connections. We then conducted a functional network analysis with clinical information from another sight. Based on the normative connectome of resting fMRI, voxel-wise correlations between the time series of bilateral VTAs and the remaining whole-brain voxels were determined for each patient with FOG, averaged throughout the normative sample and Fisher Z-transformed. The resulting patient-specific connectivity was then correlated with the %FOG-Q changes across patients at the voxel level to produce an R-map model (Fig. 1D). LOOCV was used for validation. Sex, disease duration, pre-op MoCA, pre-op HAM-A/HAM-D, LEDD reduction and UPDRS-III percent change were further included as covariates to validate the predictive model. More detailed descriptions were according to the Lead-DBS website (www.lead-dbs.org).

### Statistical analysis

All clinical variables were compared with a QQ-plot database to test the normal distribution. A paired *t*-test was used to assess the changes from baseline to follow-up only if clinical variables met the normal distribution. Otherwise, a non-parametric Wilcoxon signed-rank test was adopted. A chi-square test of independence and the Kruskal–Wallis rank-sum test were used to compare baseline differences among three or more groups. Standard deviations or interquartile ranges were used to describe the dispersion. Correlations were estimated using linear regression analysis based on the general linear model (GLM). The effect sizes were indicated as Spearman rho (*R*) correlation coefficients. A *P* < 0.05 was considered statistically significant, and the Bonferroni correction was adopted if multiple comparisons existed. All statistical and imaging analyses were performed using R Studio v2021.09.1 (R v4.1.2; PBC, Boston, MA, USA) and MATLAB R2020b (Mathworks, Natick, MA, USA).

## Results

### Clinical characteristics and stimulation settings of included patients

**Figure 2:**
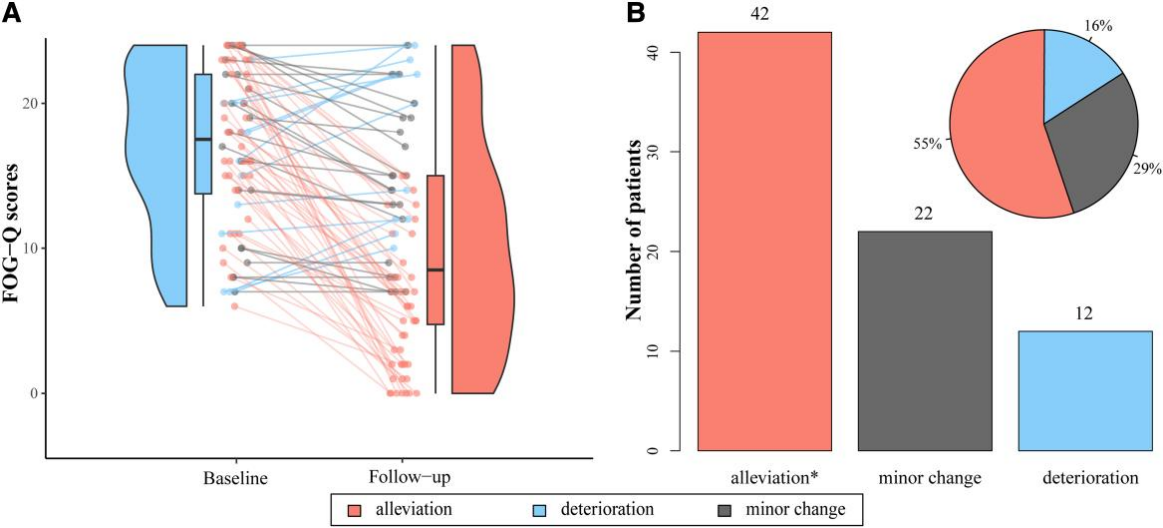
Effects of high-frequency STN-DBS on FOG. **(A)** The FOG-Q scores at baseline (blue) and follow-up (salmon). The line charts illustrate individual FOG-Q changes: salmon = FOG alleviation (FOG-Q score drops by >30%), blue = FOG deterioration (FOG-Q score increases), grey = minor change (FOG-Q score drops by <30%), and the violin plots represent overall trends (*P* < 0.001). **(B)** Among 76 FOG patients after STN-DBS, the FOG symptoms of 42 patients were alleviated (salmon), 22 patients had minor changes and 12 patients deteriorated. *The FOG symptoms of 18 patients completely disappeared after DBS.

A total of 76 Parkinson’s disease-FOG of 776 Parkinson’s disease patients were included after strictly screening the inclusion and exclusion criteria (Supplementary Fig. 1). The detailed characteristics are shown in Supplementary Table 1 and summarized in Table 1 (37 females, average age at surgery = 62.40 ± 8.66 years, 49 PINS-L301 and 27 Medtronic-3389). The average follow-up time was 12.14 ± 7.03 months. The %FOG-Q has no correlation with follow-up time (Spearman *R* = 0.2, *P* = 0.088). The pulse width, frequency and amplitude were not significantly different between right-side and left-side hemispheres (67.63 ± 11.30 versus 70.00 ± 11.43 μs, *P* = 0.602; 131.34 ± 21.24 versus 130.88 ± 19.86 Hz, *P* = 0.204; 2.36 ± 0.56 versus 2.41 ± 0.52 V, *P* = 0.926). According to the UPDRS-III, motor symptoms significantly improved (medication-off: 51.05 ± 19.75 to 22.8 ± 13.42, *P* < 0.001; medication-on: 22.32 ± 13.85 to 11.06 ± 6.82, *P* < 0.001). Pre-operative LEDD was 941.41 ± 372.74 mg as compared to post-operative 629.53 ± 252.3 mg (*P* < 0.001). Although the average FOG-Q at baseline was 16.89 ± 5.46 and significantly decreased to 10.05 ± 7.16 at follow-up (*P* < 0.001), the patient-specific changes were diverse among individuals. The average %FOG-Q change from baseline to follow-up was 37.93 ± 42.61% (42 patients were classified as alleviation, 22 patients as a minor change and 12 patients as deterioration; 18 patients in the alleviation group were further classified into the FOG-disappearance group). There was no significant difference in therapeutic stimulation parameters and pre-operative characteristics (specifically, sex, disease duration, pre-operative FOG-Q, pre-operative MMSE and pre-operative MoCA; Supplementary Table 2), which greatly minimizes the heterogeneity and bias among groups. The individual changes and overall trends are displayed in Fig. 2.

**Table 1:** Demographical features, stimulation parameters and clinical assessments of Parkinson’s disease patients with FOG

	Parkinson’s disease-FOG patients		
	Pre-op assessment	Post-op assessment	*P*-value
Demographical features			
Gender (male:female)	39:37		
Age at onset (years)	52.45 ± 9.82		
Disease duration (years)	9.79 ± 4.22		
Age at surgery (years)	62.40 ± 8.66		
MDS-UPDRS III levodopa response (%)	57.29 ± 18.35		
Clinical assessments			
Follow-up (months)		12.14 ± 7.03	
Hoehn–Yahr stage (1/1.5/2/2.5/3/4/5)	0/1/3/11/46/11/4	3/10/22/18/14/6/3	
LEDD (mg/day)	941.41 ± 372.74	629.53 ± 252.3	**<0.001**
FOG-Q	16.89 ± 5.46	10.05 ± 7.16	**<0.001**
MDS-UPDRS I	17.36 ± 7.23	11.64 ± 5.16	**<0.001**
MDS-UPDRS II	21.16 ± 7.89	11.68 ± 6.73	**<0.001**
MDS-UPDRS III (med-off)	51.05 ± 19.75	22.8 ± 13.42	**<0.001**
MDS-UPDRS III (med-on)	22.32 ± 13.85	11.06 ± 6.82	**<0.001**
MDS-UPDRS IV	10.66 ± 6.64	5.6 ± 3.53	**<0.001**
Berg balance scale (med-off)	35.87 ± 13.62	46.79 ± 10.26	**<0.001**
Berg balance scale (med-on)	47.17 ± 9.54	51.54 ± 6.28	**<0.001**
MMSE	26.11 ± 3.12	26.91 ± 3.53	0.091
MoCA score	22.61 ± 3.77	22.96 ± 4.00	0.416
HAM-D score	17.79 ± 8.02	13.17 ± 8.19	**0.003**
HAM-A score	19.08 ± 8.51	12.87 ± 8.23	**<0.001**
PDQ-39	66.18 ± 22.2	44.57 ± 26.9	**<0.001**
Stimulation parameters			
Right-side versus left-side amplitude (Hz)	2.36 ± 0.56 versus 2.41 ± 0.52		0.602
Right-side versus left-side pulse width (μs)	67.63 ± 11.30 versus 70.00 ± 11.43		0.204
Right-side versus left-side frequency (V)	131.34 ± 21.24 versus 130.88 ± 19.86		0.926

DBS, deep brain stimulation; Med, medication; pre-op, pre-operative; post-op, post-operative; PSQ-39, Parkinson’s disease questionnaire. Significant differences are bold typed.

### Electrode localization and volume of tissue activated modelling

**Figure 3:**
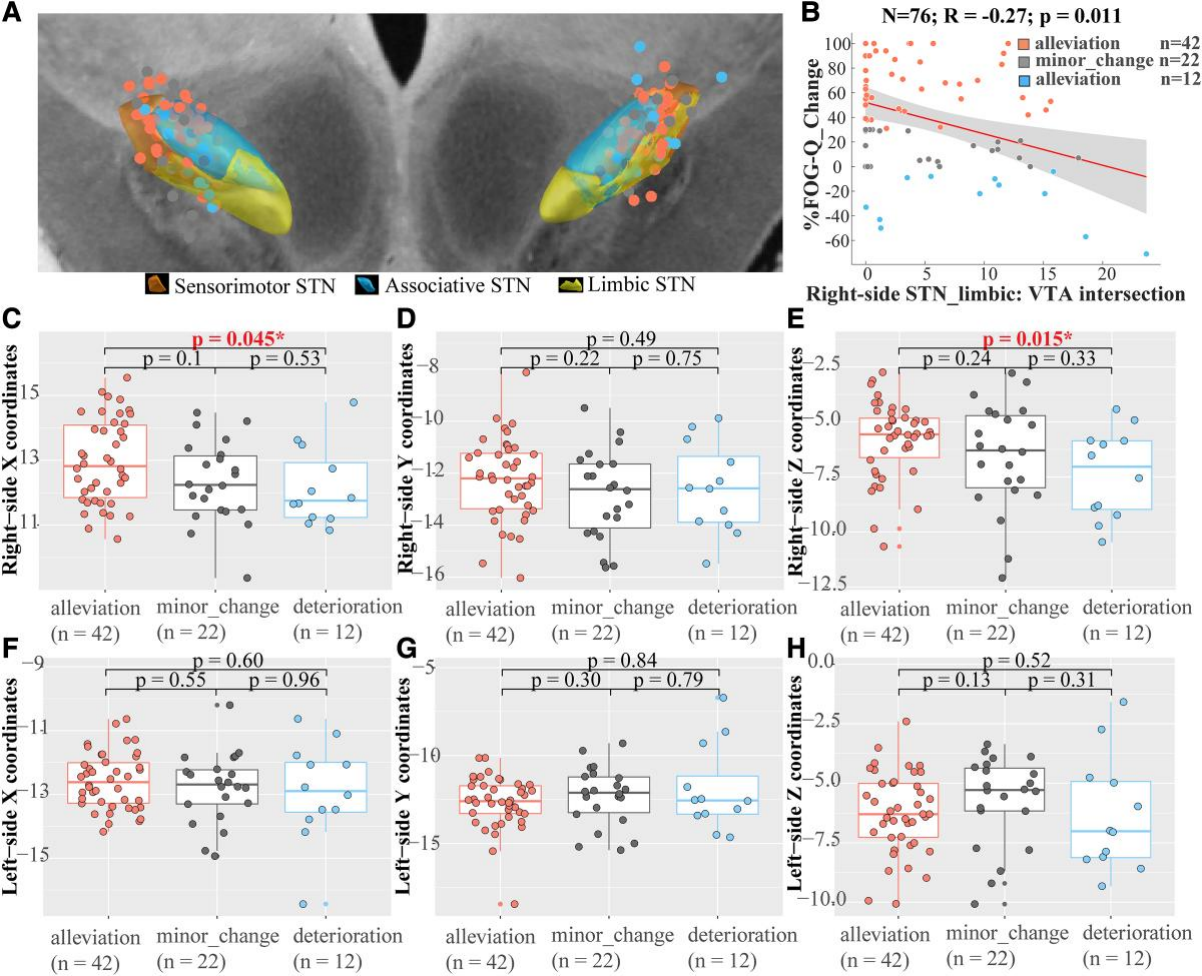
Sweet and sour stimulation contacts and VTAs. **(A)** The position of activated contacts and its relative spatial position with the STN and subregions in a 7 T 100-μm T1 MRI scan from the anterior view. **(B)** Negative correlation between the VTA and limbic STN overlap in the right hemisphere and %FOG-Q changes (*n* = 76; Spearman *R* = −0.27; *P* = 0.011); 95% confidence intervals are represented in shading areas. Three-dimensional coordinates of activated contacts in the right **(C–E)** and left **(F–H)** hemisphere among different groups. *x*, *y* and *z* coordinates represent mediolateral, anteroposterior and dorsoventral axes, respectively. Salmon dots = FOG alleviation (*n* = 42), grey dots = FOG minor change (*n* = 22), blue = FOG deterioration (*n* = 12), *significant difference.

Lead electrodes were reconstructed and localized in MNI space, and the activated contacts are shown in a 7 T 100-μm T1 MRI scan from the anterior view (Fig. 3A). By locating activated contacts as the centre, individual VTA models were calculated based on therapeutic stimulation parameters. Most of the activated contacts and calculated VTAs are inside or around the STN. Detailed coordinates of activated contacts are shown in Supplementary Table 3.

### Sweet and sour stimulation sites

#### Coordinate-level analysis

Larger *x* and *z* coordinates in the right-side hemisphere in the MNI space were shown to be significantly related to a greater %FOG-Q change (Spearman *R* = 0.27, *P* = 0.020 and Spearman *R* = 0.32, *P* = 0.005; Supplementary Table 4). Contrastingly, there are obvious differences in the activated contacts in the right-side STN between the alleviation and deterioration groups (Fig. 3C–H). Specifically, most active contacts were located at the dorsolateral two-thirds of the right STN in the FOG-alleviation group, but in the ventrocentral portion of the right STN in the FOG-deterioration group (*x* coordinates: 12.96 ± 1.31 versus 12.18 ± 1.22, *P* = 0.045; *z* coordinates: −5.81 ± 1.72 versus −7.35 ± 2.02, *P* = 0.015; the difference of *z* coordinates was still significant after Bonferroni correction; Fig. 3C and E). Eighteen patients in the alleviation group were further classified into the FOG-disappearance group. A similar outcome was generated showing that stimulation at the dorsolateral two-thirds of the right STN could lead to FOG disappearance (*P* = 0.011; Supplementary Fig. 2).

#### Volume of tissue activated-level analysis

Greater VTA and limbic STN overlap in the right hemisphere was shown to be significantly related to a worse deterioration of FOG symptom (Spearman *R* = −0.27, *P* = 0.003, still significant after Bonferroni correction; Fig. 3B), but in contrast to left hemisphere (Supplementary Fig. 3A). Moreover, the overlaps between VTA and the whole STN/sensorimotor STN/associative STN did not predict the %FOG-Q changes (Supplementary Fig. 3B–D).

#### Mapping-level analysis

**Figure 4:**
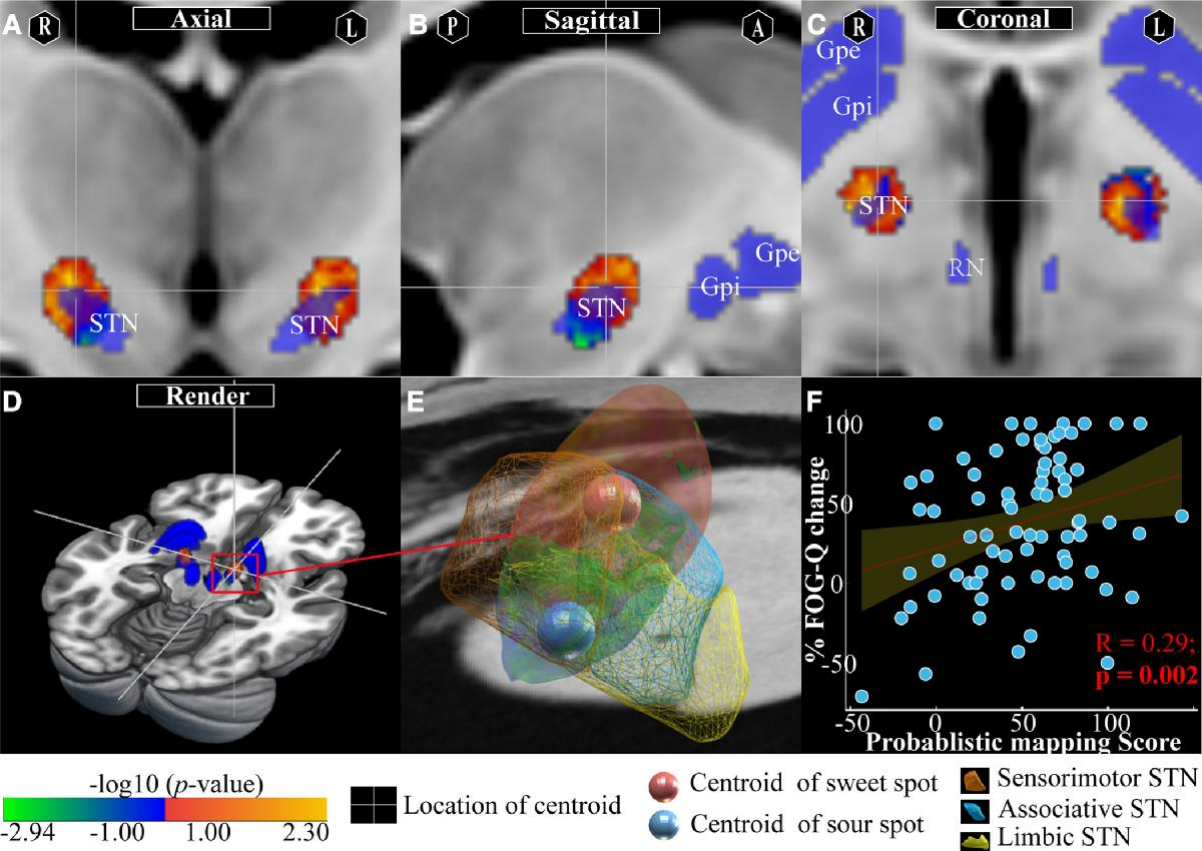
Schematic diagram of PSM. Sweet and sour spots and surrounding anatomic structures (pink regions) are shown in the axial **(A)**, sagittal **(B)**, coronal **(C)** and render **(D)** views. Hotter colours represent higher positive correlations, and colder colours represent the opposite. **(E)** The centroids of sweet and sour spots in the right hemisphere (salmon dot = centroids of sweet spots, blue dot = centroids of sour spots) and their relative spatial position with the STN and subregions. **(F)** Results of PSM analysis were validated in a LOOCV design (*n* = 76, Spearman *R* = 0.25; *P* = 0.009); 95% confidence intervals are represented by shading areas.

PSM and its relative position with surrounding anatomic structures are shown in Fig. 4A–D. Voxels at the dorsolateral two-thirds of the bilateral STN were positively associated with the %FOG-Q change, whereas the ventrocentral portion of the right STN and the internal capsule surrounding the central STN in the left hemisphere were negatively correlated with the %FOG-Q change. The values of positive and negative voxels ranged from −2.94 to 2.30. The centroids of sweet and sour spots were located at (13.7, −11.6, −4.7) and (11.7, −13.8, −8.9) in the right hemisphere (Fig. 4E), respectively, and at (−12.1, −12.6, −5.4) and (−12.7, −11.0, −5.9) in the left hemisphere (Supplementary Fig. 4), respectively. The results of PSM analysis were validated in a LOOCV design (*n* = 76, Spearman *R* = 0.29, *P* = 0.002; Fig. 4F). It remains significant even after adjusting for cohort in a combined GLM and including sex, disease duration, pre-op MoCA, pre-op HAM-A/HAM-D, LEDD decrease and UPDRS-III percent change as covariates (Spearman *R* = 0.25, *P* = 0.009).

### Structural connectivity outcomes

**Figure 5:**
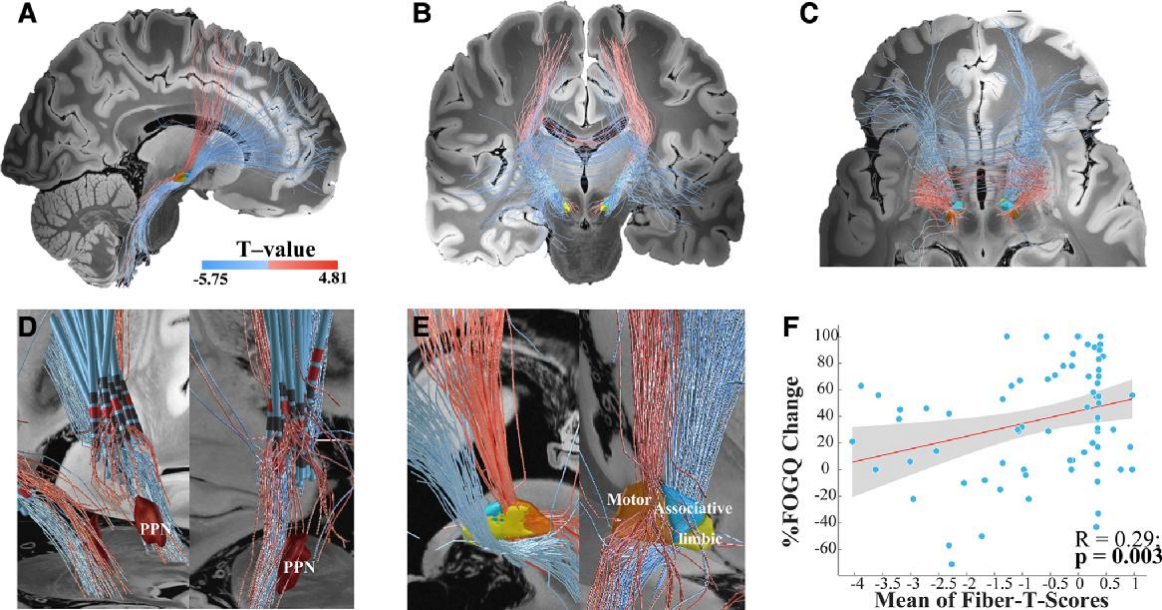
The predictive structural connectivity network model. Discriminative fibre tracts of positivity and negativity associated with the %FOG-Q changes are displayed from the sagittal **(A)**, coronal **(B)** and axial **(C)** views. The top 30% predictive tracts are displayed. Tracts in white-to-red scale represent the T-values for the positive association between selected tracts and the FOG percent change, whereas the tracts in white-to-blue represent the T-values for the negative association. Tracts cross from the VTAs in the STN to the SMA/pre-SMA were positively associated with the %FOG-Q changes, and tracts connecting the VTAs in the STN to the PFC were negatively associated with the %FOG-Q changes. **(D)** Many positive tracts extend to the MLR, in particular the PPN. **(E)** The spatial positions with the STN of positive pathways are mainly crossing through the upper dorsal and centromedial portion of the STN, while negative pathways are most crossing though or surrounding the lower ventral and centromedial of STN. **(F)** Results from this analysis were validated in a LOOCV design (*n* = 76, Spearman *R* = 0.29; *P* = 0.003); 95% confidence intervals are represented by shaded areas.

**Figure 6:**
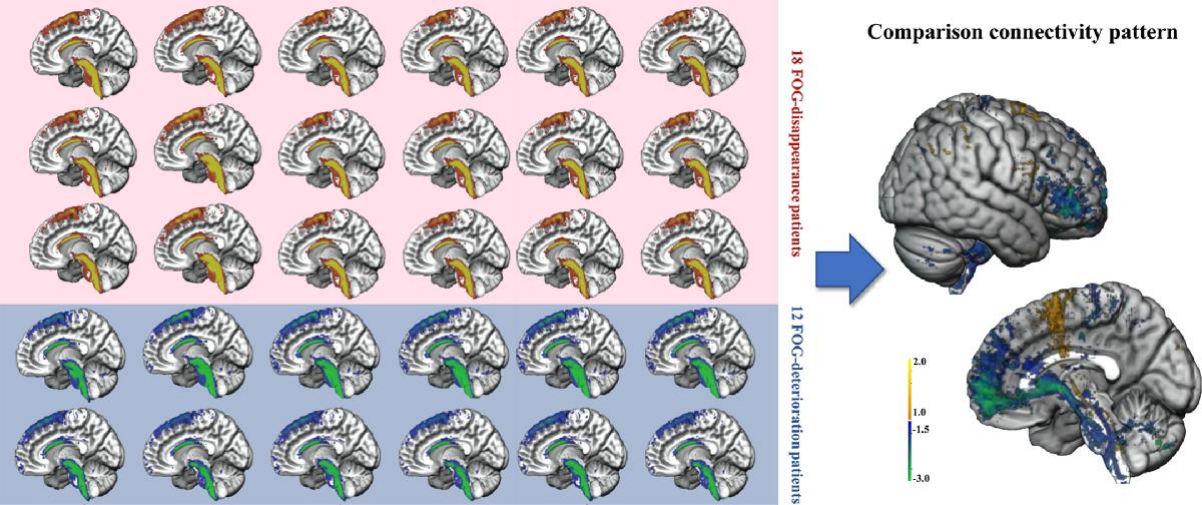
Comparative analysis of activated contacts and structural connectivity patterns between 18 FOG-disappeared patients and top 12 FOG-deteriorated patients. FOG-disappeared patients stimulated more STN–SMA/pre-SMA fibres, whereas FOG-alleviated patients stimulated more STN-PFC fibres.

Structural connectivity network results seeding from bilateral VTAs across all patients are shown in Fig. 5. The %FOG-Q changes were positively correlated with fibre tracts connecting the VTAs in the STN to the supplementary motor area and pre-SMA but negatively correlated with fibre tracts to the medial and ventrolateral prefrontal cortices (mPFC and vlPFC; Fig. 5A–C). Many positive tracts extend to the MLR/PPN (Fig. 5D). The spatial positions with STN of positive pathways were mainly crossing through the dorsolateral two-thirds of the bilateral STN, while negative pathways most crossed through the ventrocentral STN and the internal capsule surrounding the central STN (Fig. 5E). The T-values of the positive and negative tracts ranged from −5.75 to 4.81. Moreover, the LOOCV design validates this structural connectivity model (Spearman *R* = 0.29, *P* = 0.003; Fig. 5F). It remains significant after including covariates (Spearman *R* = 0.25, *P* = 0.007). To determine a more sensitive connectivity pattern, patient-specific connectivity tracts of 18 FOG-disappearance patients and 12 FOG-deterioration patients were compared (Fig. 6). In agreement with the results of the structural connectivity model, more STN–SMA/pre-SMA tracts were stimulated in the FOG-disappearance patients, whereas more STN-PFC tracts were stimulated in FOG-deterioration patients.

### Functional connectivity outcomes

**Figure 7:**
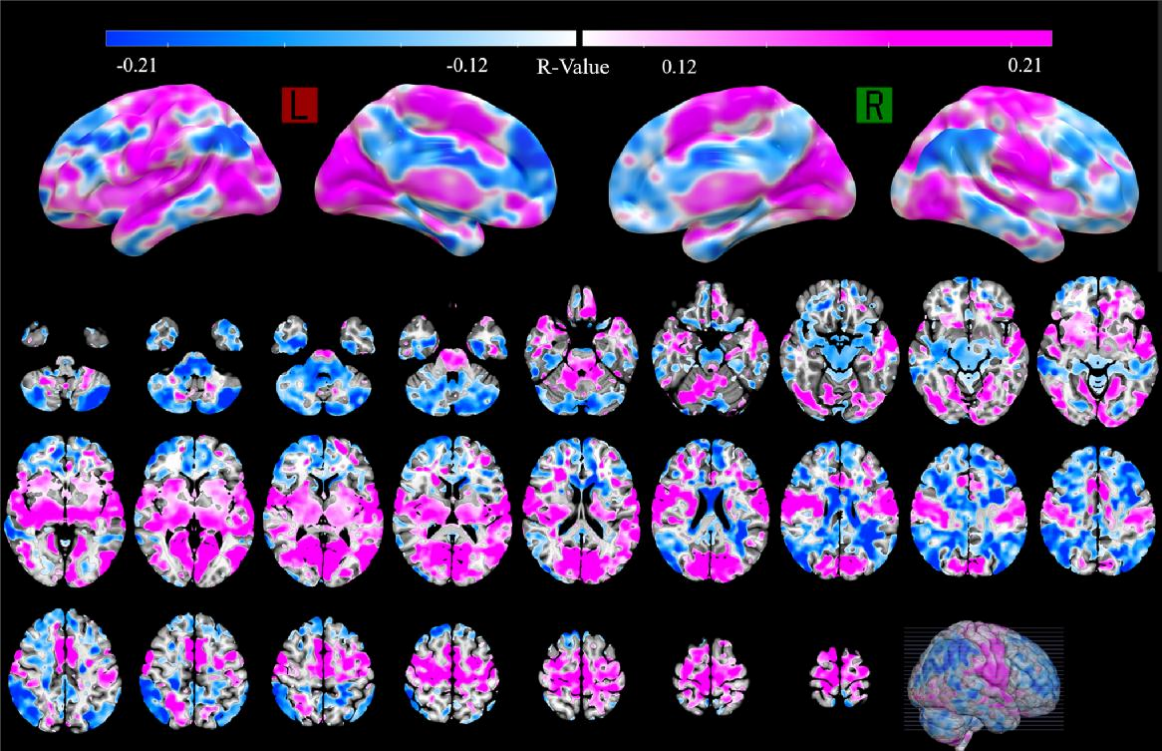
Functional connectivity network results. Hot colours represent high positive correlations, and cold colours represent negative correlations with the %FOG-Q changes. The connectivity to SMA/pre-SMA, motor cortex, basal ganglia, MLR and occipital regions is strongly correlated with FOG alleviation, but connectivity to the prefrontal regions, cerebellum and cingulate gyrus is correlating with FOG deterioration.

A profile of global functional connectivity was modelled across all included patients. The functional connectivity to the SMA/pre-SMA, motor cortex, basal ganglia, MLR, dorsal anterior cingulate cortex (dACC) and occipital regions was significantly correlated to FOG alleviation, but the connectivity to prefrontal regions, cerebellum and ventral anterior cingulate cortex (vACC) was significantly associated with FOG deterioration (Fig. 7). Although the functional network had a predictive trend, the model failed in the LOOCV analysis (Spearman *R* = 0.15, *P* = 0.098; Supplementary Fig. 5). Similar outcome was generated after including sex, disease duration, pre-op MoCA, pre-op HAM-A/HAM-D, LEDD reduction and UPDRS-III percent change as covariates (Spearman *R* = 0.14, *P* = 0.114).

## Discussion

A *post hoc* analysis of 76 Parkinson’s disease patients was performed to explore the optimal stimulation sites and connectivity network of STN-DBS treating FOG. Two main conclusions are drawn from these analyses. The dorsolateral two-thirds of the STN were considered the optimal stimulation region, whereas the ventrocentral portion of the right STN and internal capsule surrounding the left central STN were considered the sour stimulation regions. The fibre tracts connecting the supplementary motor area and pre-SMA to the dorsolateral two-thirds of the STN are recognized as the optimal bundle, while the tracts connecting mPFC and vlPFC to the ventrocentral portion of the STN and internal capsule surrounding the central STN are recognized as the sour bundle. Function connectivity results add that dACC and occipital regions may relate to alleviation, whereas the cerebellum and vACC may associate with deterioration. All of our results indicated that a broad and complex brain network was modulated during the treatment of FOG with STN-DBS.

### Specific subthalamic subregions are responsible for effective freezing of gait treatment and show bilateral asymmetry

During the process of making the pre-operative schedule, identifying the focally optimal stimulation region for specific individuals is of great importance. This pre-defined target should meet the purpose of balancing the efficacy of patients’ clinical symptoms and avoiding side effects. For motor symptoms, mainly including tremor and rigidity, the dorsolateral STN has been acknowledged as the optimal site. Severe side effects of emotional or cognitive decline, however, would occur if the electrode or stimulation regions extend out of the dorsolateral STN to the ventral or medial STN.^27^ Thus, the clinical use of STN-DBS for FOG will be encouraged by identifying the effective and aggravating regions of the STN for FOG-DBS treatment.

The association of FOG severity alleviation and the dorsolateral two-thirds of the STN is not surprising given that previous studies have mentioned the optimal targets of STN-DBS on FOG from the perspective of clinical experience. Fleury *et al*.^28^ proposed that deteriorating stimulation sites were mostly dorsal and anterior to the STN in the anterior zona incerta and Forel fields H2. Khoo *et al*.^29^ reported that the optimal stimulation sites of high- and low-frequency stimulations (HFS and LFS) are different, and the optimal regions of HFS are more lateral compared to LFS. In many electrophysiologic, imageologic and anatomic studies exploring the somatotopic organization of the STN, the lower limb-related neurons have been shown to be located at the ventrocentral portion of the dorsolateral two-thirds of the STN.^30–34^ The sweet spot and its centroid generated in our analysis overlapped with the leg region in the SMA and sensorimotor STN defined by Rodriguez-Rojas *et al*.^34^ Therefore, the local stimulation on the leg motor-related regions of the STN may influence leg functions, especially FOG in Parkinson’s disease patients. The ventrocentral portion of the STN overlaps mostly with the limbic STN and partly with the associative STN.^22,34^ The stimulation on the sour sites may add extra influences on patients’ cognition and emotion, which are also related to FOG in Parkinson’s disease patients.

Our results also observed differences in correlation between stimulation location and FOG efficacy, with significant results only for the right hemisphere. This finding can be explained by the differing volumes of the left and right STN subregions, with the left sensorimotor STN subregion being a large volume. As a result, the possibility of stimulation on the right limbic and associative STN subregions is higher.^10^ Another assumption is that the right-side STN is dominant in postural control and FOG, but the results of previous studies are still contradictory. The right-side STN has been described as the locus of inhibitory control, together with the associated SMA/pre-SMA, prefrontal cortex and locomotor areas.^35^ And, the right-side STN stimulation has been reported by many studies that achieved similar results as bilateral stimulation.^36,37^ However, Lizárraga *et al*.^38^ recently proposed that FOG-Q and axial motor signs are greatly associated with the left STN-DBS amplitude other than the right STN-DBS. No difference in post-operative FOG severity after the right and left STN-DBS has also been reported by other studies.^10,39^ Therefore, we cannot arbitrarily conclude that the right STN is a dominant DBS target for FOG. More convincing evidence should be collected to determine the potential mechanism underlying this asymmetric phenomenon.

### A broad and complex brain network that includes the motor, affective and cognitive portions was modulated, thus generating positive and negative effects

In addition to local effects, different stimulation sites also lead the diffused current towards different fibre tracts and brain pathways, modulating different networks and producing global effects on the entire brain.^40^ In our analysis, tracts connecting the SMA/pre-SMA to the dorsolateral two-thirds of the STN are responsible for FOG alleviation, while the tracts connecting the medial and ventrolateral prefrontal cortices to the ventrocentral portion of the STN and internal capsule surrounding the central STN accounted for FOG deterioration. Many positive tracts extend to the MLR/PPN. The SMA/pre-SMA, STN and MLR/PPN have been reported to have important roles during FOG occurrence and treatment.^11,13,41–43^ A set of regions of the PFC also participate in FOG occurrence and treatment.^3,10,28,44^

Using diffusing technology, the connectivity between the SMA/pre-SMA-STN-MLR/PPN pathways has been found to be significantly decreased among Parkinson’s disease-FOG patients.^37,42^ The initiation of walking and occurrence of FOG has been proposed to be greatly associated with this pathway.^45–47^ Functional MRI, PET and other cerebral imaging studies further proved this weakened connection during FOG occurrence and discovered enhanced connectivity in alleviation patients benefited from STN-DBS.^13,31,48–50^ Among studies processing walking and gait tasks, the same conclusions were reported.^51^ The increased connectivity between the STN and SMA/pre-SMA, as well as MLR/PPN, was considered to be the core mechanism of STN-DBS improving FOG in Parkinson’s disease patients. HFS on the STN has recently been proposed to selectively evoke glutamatergic but not GABAergic afferent synaptic depression.^52^ Therefore, the stimulation generates activating signals and anti-dromically activates the hyper-direct pathway,^53,54^ followed by activation of the SMA and generating an action-initiating signal. Through indirect and related pathways, this active signal sequentially inhibits gamma-aminobutyric acid (GABA) of the pallidum to the MLR, feedback to the STN, and activating the MLR/PPN. The anti-dromic activation of the SMA and hyper-direct pathway can also reduce the inhibition of the thalamus, promoting the output of walking and other motor actions to MLR/PPN.^55^ Many non-invasive stimulation studies also generated similar conclusions after local stimulations over SMA/pre-SMA and other motor cortex.^56–58^ In addition to activation effects, STN-DBS may also inhibit a feedback signal of noises that may exist throughout the entire cortex-basal-ganglia-thalamus-cortex feedback loop, restoring the pathologic brain networks towards more physiological conditions.^59^ Except for the results consistent with the fibre-filtering results, the functional connectivity added several new regions that have been reported to be associated with FOG in previous studies in which the motor cortex, dACC and occipital regions may be related to the alleviation. Activation of the dACC may play a significant role in the anti-dromic activation of the hyper-direct pathway, and occipital regions are important for visual networking. It has also been reported that the increased connectivity between the visual, motor and attention networks contribute to the alleviation of FOG.^44,60–62^

Although many studies only focused on the motor mechanism underlying FOG in isolation, recent evidence has proposed that FOG is also correlated with impairments throughout cognitive and affective networks.^63^ According to a widely accepted hypothesis, accumulating affective, cognitive and motor information results in an unresolvable response conflict, leading to the occurrence of FOG.^64^ We found that fibre tracts connecting the medial and ventrolateral prefrontal cortices to the ventrocentral portion of the STN and the internal capsule surrounding the central STN contributed to deterioration of the FOG. It was proposed that the coupling between the cognitive and affective networks was greatly correlated with ‘worse freezing severity’.^3^ Connectivity from the ventral STN to the prefrontal cortex was recently reported to be greatly associated with cognitive decline and mood change after STN-DBS or lesion.^34,65,66^ The bandwidth model we recently proposed supported the above opinions.^12^ According to this hypothesis, cognitive and motor functions compete for the information-processing resources in the motor cortex during walking. STN-DBS improves FOG by both reducing motor impairments and enhancing the overall cortical information-processing capabilities, which enables normal cognitive functioning. Stimulation over connectivity fibres between the STN and prefrontal cortex may improve decoupling between the cognitive and limbic networks, increasing cognitive and emotional load and leading to a higher possibility of FOG occurrence.

### Individualized deep brain stimulation treatment with directional leads is optimal for freezing of gait

The pre-operative schedule, operative process and post-operative programme are important steps for DBS treatment. For Parkinson’s disease patients with severe FOG symptom, electrode paths and activated contacts are recommended to cross or near the dorsolateral two-thirds of the STN in the pre-operative MRI, to stimulate sweet fibre tracts and avoid sour tracts. With the increase in stimulation amplitude and VTAs, the possibility of simulating sore sites and fibre tracts was increased due to the ring-shaped design of conventional contacts. Segmented contacts on directional leads provide current steering to particular regions for more spatially limited stimulation.^67^ To improve stimulation efficacy and reduce side effects, directional leads may be the optimal choice for individualized DBS treatment on Parkinson’s disease -FOG patients in the future.

### Limitations

A *post hoc* analysis including a set of 76 Parkinson’s disease patients was presented to determine the optimal stimulation sites and networks of STN-DBS treating FOG, but several limitations exist in this study.

First, to exclude the effect of stimulation frequency, we only included Parkinson’s disease patients with FOG whose stimulation frequency was ≥90 Hz. Many patients with low-quality imaging scans were also excluded to reach the most accurate reconstruction of DBS electrodes. Hence, only 76 of 776 patients were left.

Second, the FOG-Q scale was used as the primary tool to assess the freezing severity of included patients. Though FOG-Q has been considered as a reliable tool for assessing FOG, it cannot distinguish freezing as a result of incompatible physical, cognitive or emotional processes.^68^ Task-based gait testing would be a wonderful assessment way with a controlled design in the following studies.^12^

Third, asymmetric effects of bilateral STN-DBS, which were reported in previous studies, were also observed in this analysis.^10^ However, because each patient had just one stimulation setting, it was difficult to determine if the asymmetric findings were due to stimulation target asymmetry, anatomic asymmetry, hemisphere-dominant effects or even an unidentified mechanism. More experiments are needed to explain the asymmetric phenomenon.

Lastly, normative connectomes based on the PPMI dataset were used to process the network-level analysis, which has been proven to be a novel and great method to explore DBS treatment-related networks on the group level.^18,26^ Individual changes in patients, however, were not identified in this method. In the following studies, patient-specific diffusion scans and fMRI scans should be collected to explore the network changes at the individual level.

## Supplementary Material

fcad238_Supplementary_Data

## Data Availability

All data relevant to clinical and research information of the datasets used in this study are included in the manuscript. Supplementary materials containing individual data and active contacts’ locations are made freely available within the Open Science Framework (DOI: 10.17605/OSF.IO/2KT3). Data can be made available to qualified researchers upon reasonable request and subject to a suitable data-transfer agreement being reached.

## References

[1] Nutt JG, Bloem BR, Giladi N, et al Freezing of gait: Moving forward on a mysterious clinical phenomenon. Lancet Neurol. 2011;10(8):734–744. 21777828 10.1016/S1474-4422(11)70143-0 PMC7293393

[2] Perez-Lloret S, Negre-Pages L, Damier P, et al Prevalence, determinants, and effect on quality of life of freezing of gait in Parkinson disease. JAMA Neurol. 2014;71(7):884–890. 24839938 10.1001/jamaneurol.2014.753

[3] Ehgoetz Martens KA, Hall JM, Georgiades MJ, et al The functional network signature of heterogeneity in freezing of gait. Brain. 2018;141(4):1145–1160. 29444207 10.1093/brain/awy019

[4] Vercruysse S, Vandenberghe W, Münks L, et al Effects of deep brain stimulation of the subthalamic nucleus on freezing of gait in Parkinson's disease: A prospective controlled study. J Neurol Neurosurg Psychiatry. 2014;85(8):871–877. 24396010 10.1136/jnnp-2013-306336

[5] Karachi C, Cormier-Dequaire F, Grabli D, et al Clinical and anatomical predictors for freezing of gait and falls after subthalamic deep brain stimulation in Parkinson's disease patients. Parkinsonism Relat Disord. 2019;62:91–97. 30704853 10.1016/j.parkreldis.2019.01.021

[6] Weiss D, Schoellmann A, Fox MD, et al Freezing of gait: Understanding the complexity of an enigmatic phenomenon. Brain. 2020;143(1):14–30. 31647540 10.1093/brain/awz314 PMC6938035

[7] McNeely ME, Hershey T, Campbell MC, et al Effects of deep brain stimulation of dorsal versus ventral subthalamic nucleus regions on gait and balance in Parkinson's disease. J Neurol Neurosurg Psychiatry. 2011;82(11):1250–1255. 21478202 10.1136/jnnp.2010.232900 PMC3250990

[8] Tandra S, Kandadai RM, Peddisetty RP, et al The effect of dual tasking and deep brain stimulation frequency parameters on gait in advanced Parkinson's disease. Ann Indian Acad Neurol. 2020;23(3):308–312. 32606517 10.4103/aian.AIAN_11_19 PMC7313555

[9] Moreau C, Defebvre L, Destée A, et al STN-DBS frequency effects on freezing of gait in advanced Parkinson disease. Neurology. 2008;71(2):80–84. 18420482 10.1212/01.wnl.0000303972.16279.46

[10] Temiz G, Santin MDN, Olivier C, et al Freezing of gait depends on cortico-subthalamic network recruitment following STN-DBS in PD patients. Parkinsonism Relat Disord. 2022;104:49–57. 36242900 10.1016/j.parkreldis.2022.10.002

[11] Strelow JN, Baldermann JC, Dembek TA, et al Structural connectivity of subthalamic nucleus stimulation for improving freezing of gait. J Parkinsons Dis. 2022;12(4):1251–1267. 35431262 10.3233/JPD-212997

[12] Yin Z, Zhu G, Liu Y, et al Cortical phase-amplitude coupling is key to the occurrence and treatment of freezing of gait. Brain. 2022;145(7):2407–2421. 35441231 10.1093/brain/awac121 PMC9337810

[13] Weiss PH, Herzog J, Pötter-Nerger M, et al Subthalamic nucleus stimulation improves parkinsonian gait via brainstem locomotor centers. Mov Disord. 2015;30(8):1121–1125. 25914247 10.1002/mds.26229

[14] Xie T, Kang UJ, Warnke P. Effect of stimulation frequency on immediate freezing of gait in newly activated STN DBS in Parkinson's disease. J Neurol Neurosurg Psychiatry. 2012;83(10):1015–1017. 22696586 10.1136/jnnp-2011-302091

[15] Giladi N, Tal J, Azulay T, et al Validation of the freezing of gait questionnaire in patients with Parkinson's disease. Mov Disord. 2009;24(5):655–661. 19127595 10.1002/mds.21745

[16] Schlenstedt C, Shalash A, Muthuraman M, et al Effect of high-frequency subthalamic neurostimulation on gait and freezing of gait in Parkinson's disease: A systematic review and meta-analysis. Eur J Neurol. 2017;24(1):18–26. 27766724 10.1111/ene.13167

[17] Parkinson Progression Marker Initiative . The Parkinson progression marker initiative (PPMI). Prog Neurobiol. 2011;95(4):629–635. 21930184 10.1016/j.pneurobio.2011.09.005 PMC9014725

[18] Horn A, Reich M, Vorwerk J, et al Connectivity predicts deep brain stimulation outcome in Parkinson disease. Ann Neurol. 2017;82(1):67–78. 28586141 10.1002/ana.24974 PMC5880678

[19] Avants BB, Tustison NJ, Song G, et al A reproducible evaluation of ANTs similarity metric performance in brain image registration. Neuroimage. 2011;54(3):2033–2044. 20851191 10.1016/j.neuroimage.2010.09.025 PMC3065962

[20] Schönecker T, Kupsch A, Kühn AA, et al Automated optimization of subcortical cerebral MR imaging-atlas coregistration for improved postoperative electrode localization in deep brain stimulation. AJNR Am J Neuroradiol. 2009;30(10):1914–1921. 19713324 10.3174/ajnr.A1741 PMC7051288

[21] Husch A, V Petersen M, Gemmar P, Goncalves J, Hertel F. PaCER—A fully automated method for electrode trajectory and contact reconstruction in deep brain stimulation. Neuroimage Clin. 2018;17:80–89. 29062684 10.1016/j.nicl.2017.10.004 PMC5645007

[22] Ewert S, Plettig P, Li N, et al Toward defining deep brain stimulation targets in MNI space: A subcortical atlas based on multimodal MRI, histology and structural connectivity. Neuroimage. 2018;170:271–282. 28536045 10.1016/j.neuroimage.2017.05.015

[23] Horn A, Li N, Dembek TA, et al Lead-DBS v2: Towards a comprehensive pipeline for deep brain stimulation imaging. Neuroimage. 2019;184:293–316. 30179717 10.1016/j.neuroimage.2018.08.068 PMC6286150

[24] Vorwerk J, Oostenveld R, Piastra MC, et al The FieldTrip-SimBio pipeline for EEG forward solutions. Biomed Eng Online. 2018;17(1):37. 29580236 10.1186/s12938-018-0463-y PMC5870695

[25] Elias GJB, Boutet A, Joel SE, et al Probabilistic mapping of deep brain stimulation: Insights from 15 years of therapy. Ann Neurol. 2021;89(3):426–443. 33252146 10.1002/ana.25975

[26] Li N, Hollunder B, Baldermann JC, et al A unified functional network target for deep brain stimulation in obsessive-compulsive disorder. Biol Psychiatry. 2021;90(10):701–713. 34134839 10.1016/j.biopsych.2021.04.006

[27] Irmen F, Horn A, Mosley P, et al Left prefrontal connectivity links subthalamic stimulation with depressive symptoms. Ann Neurol. 2020;87(6):962–975. 32239535 10.1002/ana.25734

[28] Fleury V, Pollak P, Gere J, et al Subthalamic stimulation may inhibit the beneficial effects of levodopa on akinesia and gait. Mov Disord. 2016;31(9):1389–1397. 26887333 10.1002/mds.26545

[29] Khoo HM, Kishima H, Hosomi K, et al Low-frequency subthalamic nucleus stimulation in Parkinson's disease: A randomized clinical trial. Mov Disord. 2014;29(2):270–274. 24449169 10.1002/mds.25810

[30] Romanelli P, Heit G, Hill BC, et al Microelectrode recording revealing a somatotopic body map in the subthalamic nucleus in humans with Parkinson disease. J Neurosurg. 2004;100(4):611–618. 15070113 10.3171/jns.2004.100.4.0611

[31] Rodriguez-Oroz MC, Rodriguez M, Guridi J, et al The subthalamic nucleus in Parkinson's disease: Somatotopic organization and physiological characteristics. Brain. 2001;124(Pt 9):1777–1790. 11522580 10.1093/brain/124.9.1777

[32] Abosch A, Hutchison WD, Saint-Cyr JA, et al Movement-related neurons of the subthalamic nucleus in patients with Parkinson disease. J Neurosurg. 2002;97(5):1167–1172. 12450039 10.3171/jns.2002.97.5.1167

[33] Alimonti D, Donati R, Foresti C, et al Somatotopic organization of STN and DBS implications in Parkinson's disease—A case report of a woman “halved” by DBS stimulation. Brain Stimul. 2020;13(5):1384–1386. 32712340 10.1016/j.brs.2020.07.014

[34] Rodriguez-Rojas R, Pineda-Pardo JA, Mañez-Miro J, et al Functional topography of the human subthalamic nucleus: Relevance for subthalamotomy in Parkinson's disease. Mov Disord. 2022;37(2):279–290. 34859498 10.1002/mds.28862

[35] Aron AR, Robbins TW, Poldrack RA. Inhibition and the right inferior frontal cortex: One decade on. Trends Cogn Sci. 2014;18(4):177–185. 24440116 10.1016/j.tics.2013.12.003

[36] Lizarraga KJ, Jagid JR, Luca CC. Comparative effects of unilateral and bilateral subthalamic nucleus deep brain stimulation on gait kinematics in Parkinson’s disease: A randomized, blinded study. J Neurol. 2016;263(8):1652–1656. 27278062 10.1007/s00415-016-8191-3

[37] Fling BW, Cohen RG, Mancini M, et al Asymmetric pedunculopontine network connectivity in parkinsonian patients with freezing of gait. Brain. 2013;136(Pt 8):2405–2418. 23824487 10.1093/brain/awt172 PMC3722352

[38] Lizárraga KJ, Gnanamanogaran B, Al-Ozzi TM, et al Lateralized subthalamic stimulation for axial dysfunction in Parkinson's disease: A randomized trial. Mov Disord. 2022;37(5):1079–1087. 35156734 10.1002/mds.28953

[39] Castrioto A, Meaney C, Hamani C, et al The dominant-STN phenomenon in bilateral STN DBS for Parkinson's disease. Neurobiol Dis. 2011;41(1):131–137. 20826212 10.1016/j.nbd.2010.08.029

[40] Sobesky L, Goede L, Odekerken VJJ, et al Subthalamic and pallidal deep brain stimulation: Are we modulating the same network? Brain. 2022;145(1):251–262. 34453827 10.1093/brain/awab258

[41] Bardakan MM, Fink GR, Zapparoli L, et al Imaging the neural underpinnings of freezing of gait in Parkinson's disease. Neuroimage Clin. 2022;35:103123. 35917720 10.1016/j.nicl.2022.103123 PMC9421505

[42] Hall JM, Shine JM, Ehgoetz Martens KA, et al Alterations in white matter network topology contribute to freezing of gait in Parkinson's disease. J Neurol. 2018;265(6):1353–1364. 29616302 10.1007/s00415-018-8846-3

[43] Pietracupa S, Suppa A, Upadhyay N, et al Freezing of gait in Parkinson's disease: Gray and white matter abnormalities. J Neurol. 2018;265(1):52–62. 29128929 10.1007/s00415-017-8654-1

[44] Canu E, Agosta F, Sarasso E, et al Brain structural and functional connectivity in Parkinson's disease with freezing of gait. Hum Brain Mapp. 2015;36(12):5064–5078. 26359798 10.1002/hbm.22994 PMC6869160

[45] Peterson DS, Smulders K, Mancini M, et al Relating response inhibition, brain connectivity, and freezing of gait in people with Parkinson's disease. J Int Neuropsychol Soc. 2021;27(7):733–743. 33292899 10.1017/S135561772000123X PMC8187475

[46] Bharti K, Suppa A, Pietracupa S, et al Abnormal cerebellar connectivity patterns in patients with Parkinson's disease and freezing of gait. Cerebellum. 2019;18(3):298–308. 30392037 10.1007/s12311-018-0988-4

[47] Nachev P, Wydell H, O'Neill K, et al The role of the pre-supplementary motor area in the control of action. Neuroimage. 2007;36(Suppl 2(3-3)):T155–T163. 17499162 10.1016/j.neuroimage.2007.03.034 PMC2648723

[48] Myers PS, McNeely ME, Pickett KA, et al Effects of exercise on gait and motor imagery in people with Parkinson disease and freezing of gait. Parkinsonism Relat Disord. 2018;53:89–95. 29754837 10.1016/j.parkreldis.2018.05.006 PMC6120800

[49] Ballanger B, Lozano AM, Moro E, et al Cerebral blood flow changes induced by pedunculopontine nucleus stimulation in patients with advanced Parkinson's disease: A [(15)O] H2O PET study. Hum Brain Mapp. 2009;30(12):3901–3909. 19479730 10.1002/hbm.20815 PMC6871082

[50] Tard C, Delval A, Devos D, et al Brain metabolic abnormalities during gait with freezing in Parkinson's disease. Neuroscience. 2015;307:281–301. 26341909 10.1016/j.neuroscience.2015.08.063

[51] Snijders AH, Leunissen I, Bakker M, et al Gait-related cerebral alterations in patients with Parkinson's disease with freezing of gait. Brain. 2011;134(Pt 1):59–72. 21126990 10.1093/brain/awq324

[52] Steiner LA, Barreda Tomás FJ, Planert H, et al Connectivity and dynamics underlying synaptic control of the subthalamic nucleus. J Neurosci. 2019;39(13):2470–2481. 30700533 10.1523/JNEUROSCI.1642-18.2019 PMC6435833

[53] McIntyre CC, Hahn PJ. Network perspectives on the mechanisms of deep brain stimulation. Neurobiol Dis. 2010;38(3):329–337. 19804831 10.1016/j.nbd.2009.09.022 PMC2862840

[54] Oswal A, Cao C, Yeh C-H, et al Neural signatures of hyperdirect pathway activity in Parkinson’s disease. Nat Commun. 2021;12(1):5185. 34465771 10.1038/s41467-021-25366-0 PMC8408177

[55] Ebersbach G, Moreau C, Gandor F, et al Clinical syndromes: Parkinsonian gait. Mov Disord. 2013;28(11):1552–1559. 24132843 10.1002/mds.25675

[56] Mi TM, Garg S, Ba F, et al Repetitive transcranial magnetic stimulation improves Parkinson's freezing of gait via normalizing brain connectivity. NPJ Parkinsons Dis. 2020;6:16. 32699818 10.1038/s41531-020-0118-0 PMC7368045

[57] Manor B, Dagan M, Herman T, et al Multitarget transcranial electrical stimulation for freezing of gait: A randomized controlled trial. Mov Disord. 2021;36(11):2693–2698. 34406695 10.1002/mds.28759

[58] Dagan M, Herman T, Harrison R, et al Multitarget transcranial direct current stimulation for freezing of gait in Parkinson's disease. Mov Disord. 2018;33(4):642–646. 29436740 10.1002/mds.27300 PMC5964604

[59] Eusebio A, Thevathasan W, Doyle Gaynor L, et al Deep brain stimulation can suppress pathological synchronisation in parkinsonian patients. J Neurol Neurosurg Psychiatry. 2011;82(5):569–573. 20935326 10.1136/jnnp.2010.217489 PMC3072048

[60] Bharti K, Suppa A, Pietracupa S, et al Aberrant functional connectivity in patients with Parkinson's disease and freezing of gait: A within- and between-network analysis. Brain Imaging Behav. 2020;14(5):1543–1554. 30887415 10.1007/s11682-019-00085-9

[61] Gallea C, Wicki B, Ewenczyk C, et al Antisaccade, a predictive marker for freezing of gait in Parkinson's disease and gait/gaze network connectivity. Brain. 2021;144(2):504–514. 33279957 10.1093/brain/awaa407

[62] Bejjani BP, Gervais D, Arnulf I, et al Axial parkinsonian symptoms can be improved: The role of levodopa and bilateral subthalamic stimulation. J Neurol Neurosurg Psychiatry. 2000;68(5):595–600. 10766889 10.1136/jnnp.68.5.595 PMC1736917

[63] Szeto JY, O'Callaghan C, Shine JM, et al The relationships between mild cognitive impairment and phenotype in Parkinson's disease. NPJ Parkinsons Dis. 2015;1:15015. 28725684 10.1038/npjparkd.2015.15 PMC5516553

[64] Nieuwboer A, Giladi N. Characterizing freezing of gait in Parkinson's disease: Models of an episodic phenomenon. Mov Disord. 2013;28(11):1509–1519. 24132839 10.1002/mds.25683

[65] Reich MM, Hsu J, Ferguson M, et al A brain network for deep brain stimulation induced cognitive decline in Parkinson's disease. Brain. 2022;145(4):1410–1421. 35037938 10.1093/brain/awac012 PMC9129093

[66] Kuhn J, Wong JK, Okun MS, et al Connectomic imaging to predict and prevent cognitive decline after subthalamic DBS: Next steps. Brain. 2022;145(4):1204–1206. 35608893 10.1093/brain/awac101

[67] Schüpbach M, Chabardes S, Matthies C, et al Directional leads for deep brain stimulation: Opportunities and challenges. Mov Disord. 2017;32(10):1371–1375. 28843016 10.1002/mds.27096

[68] Shine JM, Moore ST, Bolitho SJ, et al Assessing the utility of freezing of gait questionnaires in Parkinson's disease. Parkinsonism Relat Disord. 2012;18(1):25–29. 21872523 10.1016/j.parkreldis.2011.08.002

