# Supplementary material for: Optimal subthalamic stimulation sites and related networks for freezing of gait in Parkinson’s disease
Source: Brain Commun. 2023 Sep 4;5(5):fcad238. doi: 10.1093/braincomms/fcad238 (PMC10493641; doi:10.1093/braincomms/fcad238)
Supplement: fcad238_Supplementary_Data [file fcad238_supplementary_data.docx]

**Supplementary Material for:**

**Optimal subthalamic stimulation sites and related networks for freezing of gait in Parkinson’s disease**

**Contents:**

**Number of words in** **supplementary methods:** 476

**Number of supplementary figures:** 5

**Number of supplementary tables:** 4

**Methods**

**Surgical and programming procedure**

A 3T magnetic resonance image (MRI) with high resolution (1 * 1 * 1 mm^3^) of every patient’s head was obtained 1 day before the surgery. Then, a thin-layer computed tomography (CT) head scan (spacing 0.625 mm) was conducted with a Leksell stereotactic frame mounted on the head on the day of the surgical procedure. The CT and MRI images were merged to determine the implantation trajectory plan and localize the bilateral STN contacts. Generally, the STN target coordinates were 1–3 mm posterior to the MCP, 11–14 mm lateral to AC‐PC, and 4–6 mm below the inter‐commissural line. DBS electrode implantation was performed under local anesthesia, using a Leksell microstereotactic system (Elekta Instrument AB, Stockholm, Sweden).

Intraoperative microelectrode recording measuring the length of the DBS trajectory in the STN and macro-stimulation tests were conducted for trajectory selection. The tip of the microelectrode was guided to the dorsolateral portions of the STN based on the implantation trajectory plan. Microelectrode recording started 15 mm above the predefined target and the recorded neuronal activity was used to help define the boundary of STN during surgery. The side-specific effects of intraoperative stimulations were also used to confirm the optimal location. If intraoperative stimulation induces adverse reactions in patients, the position of the electrode would be readjusted until satisfactory effects were achieved.

After the target location was confirmed, the microelectrode was pulled out and a quadripolar electrode (PINS-L301 or Medtronic-3389) was implanted along the microelectrode trajectory to the position of STN target. The electrodes were then connected to an implantable pulse generator (IPG) implanted in the subclavicular area under general anesthesia. Next, post‐operative CT was performed to exclude intracranial hemorrhage and to verify the exact location of the electrodes by merging them with the preoperative MR images.

The IPG was turned on 1 month after the operation. Following the surgery, each patient underwent a regular adjustment of stimulation settings and medication until optimal control of symptoms was established. Typically, the patients’ improvement stabilized 6 months after surgery. Patients had visited at least every 6 months for clinical assessments and adjustment of stimulation settings and medication. All post‐operative adjustment of DBS parameter settings was performed while subjects were in the off‐medication and on-medication state after at least 12 hours without taking any dopaminergic medications.

**Processes generating normative connectomes**

For DTI scans, whole brain tractography fiber sets were calculated using a generalized q-sampling imaging algorithm as implemented in DSI Studio. Sampling was performed within a white matter mask that was defined using the unified segmentation approach on T2-weighted structural acquisitions and that was co-registered to the b0 volume using SPM12^1^. In each subject, 200000 fibers were sampled. Fiber tracts were then transformed into MNI space^2-4^. For rs-fMRI scans, processing included global signal regression and spatial smoothing at 6mm full width at half maximum as previously described^5^.

**Supplementary references**

[1] Horn A, Kühn AA, Merkl A, Shih L, Alterman R, Fox M. Probabilistic conversion of neurosurgical DBS electrode coordinates into MNI space. NeuroImage 2017;150:395-404.

[2] Horn A, Ostwald D, Reisert M, Blankenburg F. The structural-functional connectome and the default mode network of the human brain. NeuroImage 2014;102 Pt 1:142-51.

[3] Horn A, Blankenburg F. Toward a standardized structural-functional group connectome in MNI space. NeuroImage 2016;124(Pt A):310-22.

[4] Horn A, Neumann WJ, Degen K, Schneider GH, Kühn AA. Toward an electrophysiological "sweet spot" for deep brain stimulation in the subthalamic nucleus. Human brain mapping 2017;38(7):3377-90.

[5] Yeo BT, Krienen FM, Sepulcre J, Sabuncu MR, Lashkari D, Hollinshead M, et al. The organization of the human cerebral cortex estimated by intrinsic functional connectivity. Journal of neurophysiology 2011;106(3):1125-65.

**Supplementary Figures**


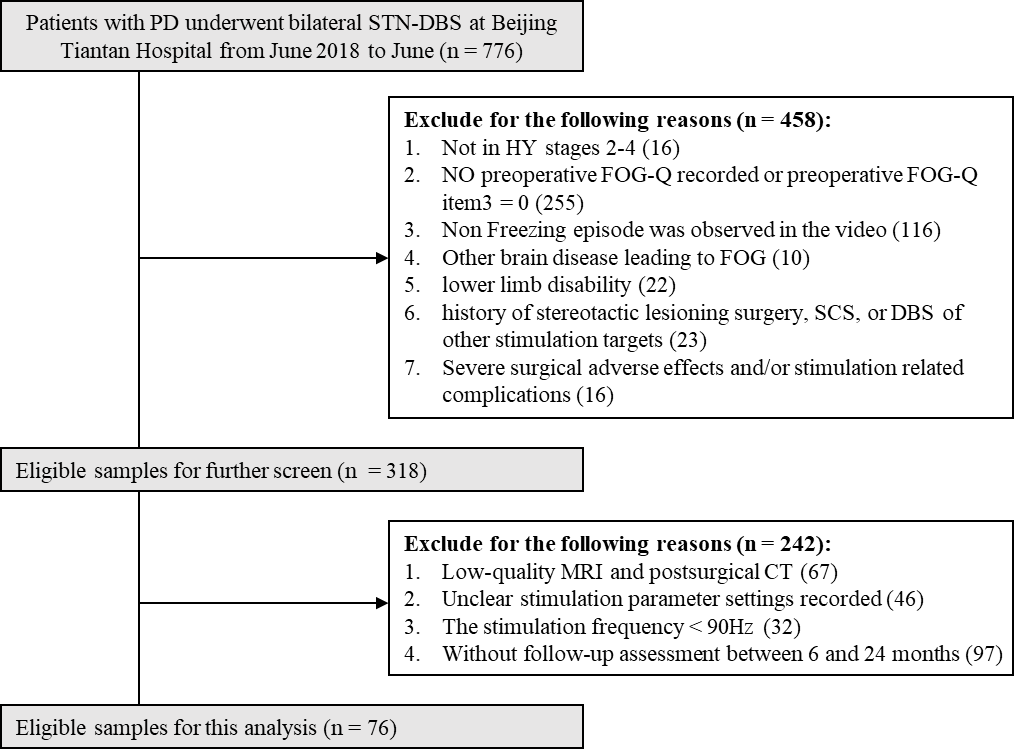


**Supplementary Figure 1:** The workflow of participants’ identification in this analysis.


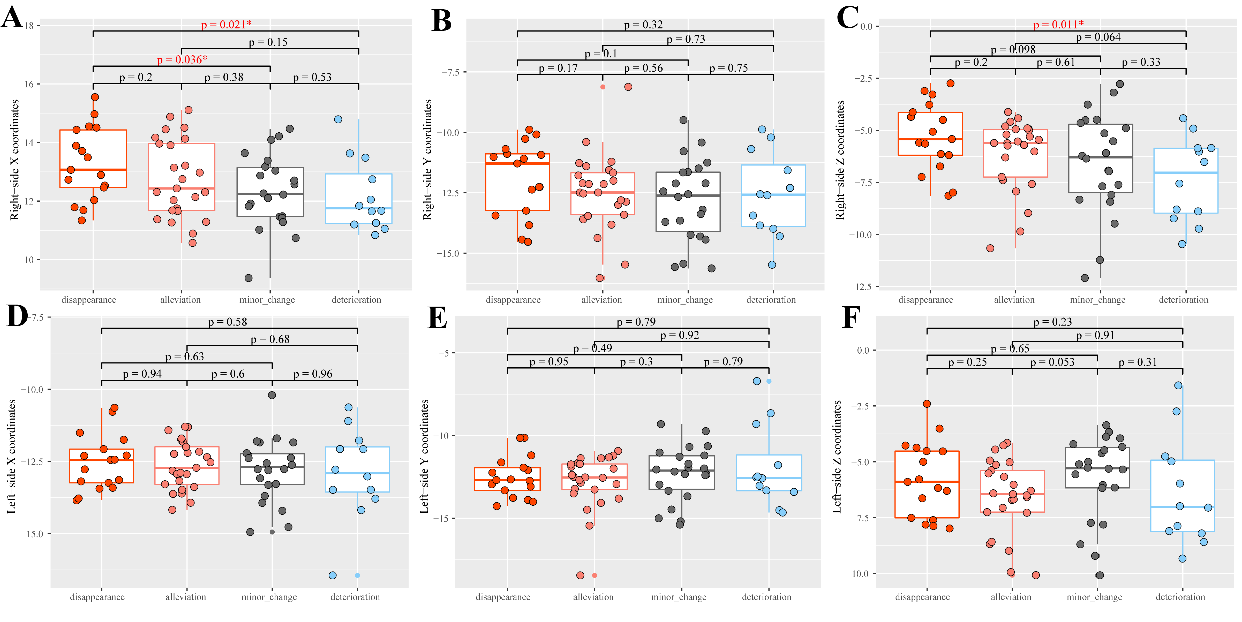


**Supplementary Figure 2:** Sensitivity analysis results of sweet and sour stimulation contacts (For A-F figures, total N = 76; N{disappearance} : N{alleviation} : N{minor_change} : N{deterioration} = 18 : 24 : 22 : 12)

.


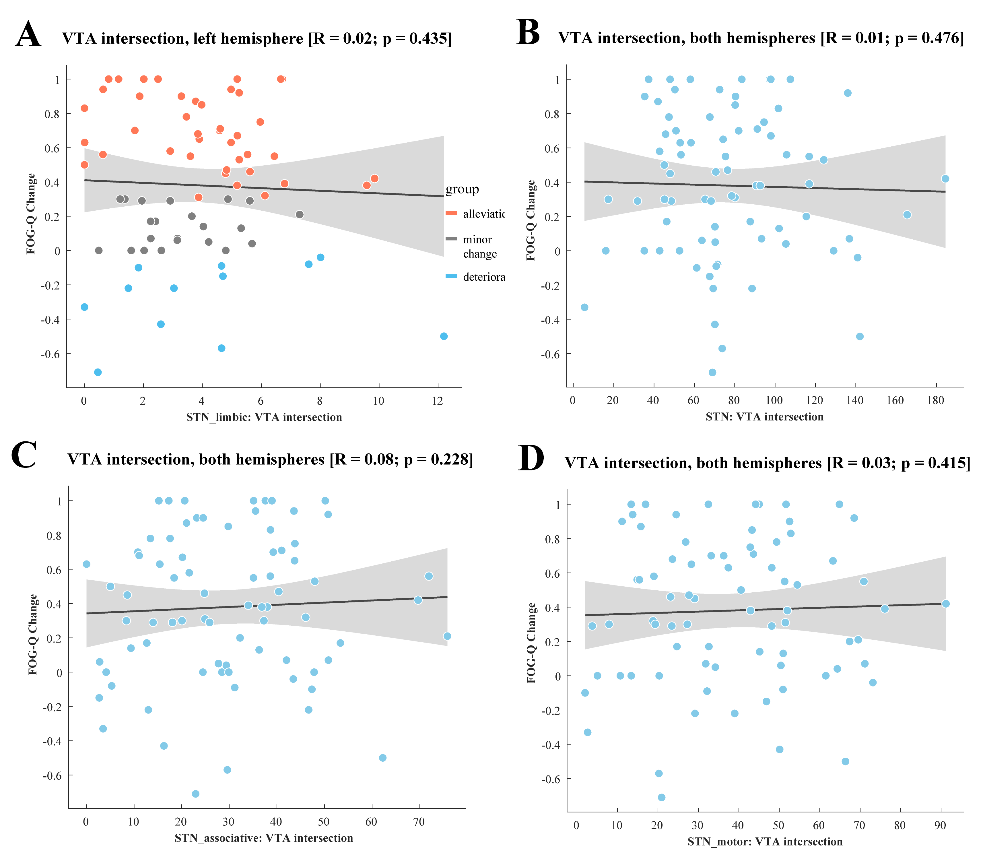


**Supplementary Figure 3:** Correlations between the VTA with STN/subregions overlap and FOG-Q percent change (N = 76). 95% confidence intervals are represented in shading areas.


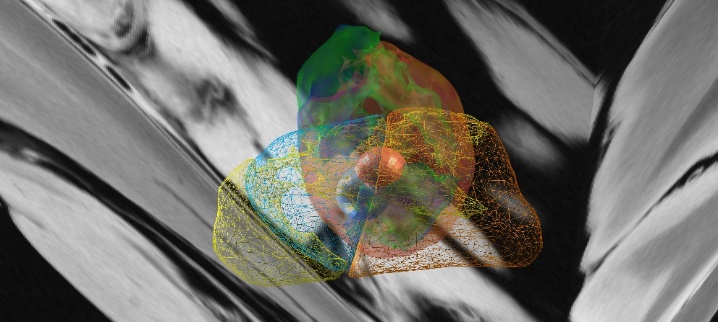


**Supplementary Figure4:** The centroids of sweet & sour spots in the left hemisphere (salmon dot = centroids of sweet spots, blue dot = centroid of sour spots), and their relative spatial position with STN & subregions.


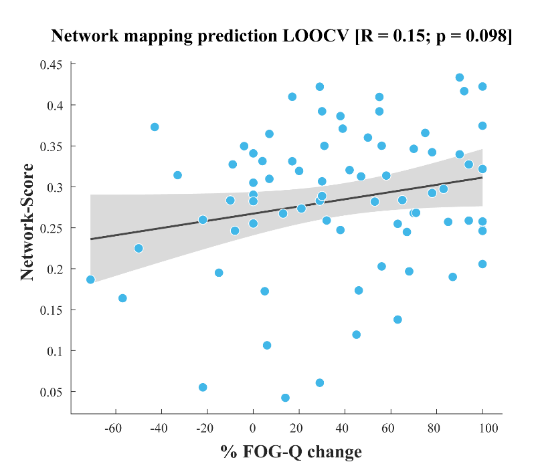


**Supplementary Figure 5:** Results of leave-one-out cross-validation analysis for functional connectivity model (N = 76, *R* = 0.15, *p* = 0.098). 95% confidence intervals are represented by shading areas.

**Supplementary Tables**

**Supplementary Table 1** Details of included FOG patients

|  |  |  |  | **Contacts** | | **Stimulation parameters** | | |  |  |  |  |  |
| --- | --- | --- | --- | --- | --- | --- | --- | --- | --- | --- | --- | --- | --- |
| **Patient ID** | **Age** | **Sex** | **Lead Type** | **Anode (+)** | **Cathode (-)** | **Pulse Width(****μs)** | **Frequency（Hz）** | **Amplitude (V/mA)** | **Pre-operation FOG-Q** | **Pre-operation FOG-Q** | **%FOG-Q change (%)** | **Change type** |  |
| 1 | 50 | F | PINS, L301 | Case | 3 | 60 | 130 | 2.3 | 15 | 7 | 53.33 | alleviation |  |
|  |  |  |  |  | 7 | 60 | 130 | 2.3 |  |  |  |  |  |
| 2 | 58 | M | Medtronic, 3389S | Case | 2 | 80 | 120 | 2.7 | 23 | 7 | 69.57 | alleviation |  |
|  |  |  |  |  | 9 | 70 | 120 | 1.7 |  |  |  |  |  |
| 3 | 60 | F | PINS, L301 | Case | 3, 4 | 80 | 140 | 2.5 | 24 | 15 | 37.5 | alleviation |  |
|  |  |  |  |  | 7, 8 | 90 | 140 | 3 |  |  |  |  |  |
| 4 | 65 | M | PINS, L301 | Case | 3 | 80 | 90 | 3 | 18 | 15 | 16.67 | minor change |  |
|  |  |  |  |  | 6 | 70 | 90 | 3 |  |  |  |  |  |
| 5 | 73 | M | PINS, L301 | Case | 3 | 60 | 150 | 2.5 | 8 | 8 | 0 | minor change |  |
|  |  |  |  |  | 7 | 60 | 150 | 2.5 |  |  |  |  |  |
| 6 | 77 | M | PINS, L301 | Case | 3 | 80 | 155 | 2.5 | 20 | 14 | 30 | minor change |  |
|  |  |  |  |  | 8 | 60 | 155 | 1.9 |  |  |  |  |  |
| 7 | 51 | F | Medtronic, 3389S | Case | 2 | 70 | 155 | 2.2 | 11 | 5 | 54.55 | alleviation |  |
|  |  |  |  |  | 10 | 70 | 155 | 2.65 |  |  |  |  |  |
| 8 | 67 | F | PINS, L301 | Case | 2 | 60 | 135 | 1.9 | 11 | 0 | 100 | alleviation |  |
|  |  |  |  |  | 6- | 60 | 135 | 1.9 |  |  |  |  |  |
| 9 | 56 | F | PINS, L301 | Case | 4 | 70 | 170 | 0.35 | 7 | 7 | 0 | minor change |  |
|  |  |  |  |  | 8 | 100 | 170 | 3.1 |  |  |  |  |  |
| 10 | 67 | F | Medtronic, 3389S | Case | 2 | 80 | 130 | 2 | 23 | 0 | 100 | alleviation |  |
|  |  |  |  |  | 10 | 80 | 130 | 2.1 |  |  |  |  |  |
| 11 | 73 | M | PINS, L301 | Case | 3 | 60 | 150 | 2.35 | 24 | 17 | 29.17 | minor change |  |
|  |  |  |  |  | 6 | 90 | 150 | 3 |  |  |  |  |  |
| 12 | 41 | F | PINS, L301 | Case | 1, 3 | 80 | 120 | 2.3 | 24 | 19 | 20.83 | minor change |  |
|  |  |  |  |  | 6, 8 | 70 | 120 | 2.5 |  |  |  |  |  |
| 13 | 72 | F | PINS, L301 | Case | 4 | 60 | 130 | 2 | 20 | 6 | 70 | alleviation |  |
|  |  |  |  |  | 8 | 60 | 130 | 2.3 |  |  |  |  |  |
| 14 | 39 | M | PINS, L301 | Case | 3 | 60 | 140 | 2 | 10 | 1 | 90 | alleviation |  |
|  |  |  |  |  | 7 | 70 | 140 | 2.7 |  |  |  |  |  |
| 15 | 71 | F | Medtronic, 3389S | Case | 3 | 70 | 130 | 1.75 | 16 | 24 | -50 | deterioration |  |
|  |  |  |  |  | 6, 7 | 70 | 130 | 2.95 |  |  |  |  |  |
| 16 | 69 | F | PINS, L301 | Case | 4 | 60 | 143 | 2 | 18 | 8 | 55.56 | alleviation |  |
|  |  |  |  |  | 8 | 70 | 143 | 2 |  |  |  |  |  |
| 17 | 46 | F | Medtronic, 3389S | Case | 2 | 70 | 90 | 2.9 | 24 | 15 | 37.5 | alleviation |  |
|  |  |  |  |  | 10 | 80 | 90 | 2.9 |  |  |  |  |  |
| 18 | 62 | F | PINS, L301 | Case | 3, 4 | 60 | 130 | 1.5 | 23 | 5 | 78.26 | alleviation |  |
|  |  |  |  |  | 8 | 90 | 130 | 2.8 |  |  |  |  |  |
| 19 | 70 | F | Medtronic, 3389S | Case | 2 | 60 | 140 | 2.2 | 18 | 11 | 38.89 | alleviation |  |
|  |  |  |  |  | 10 | 60 | 140 | 2.5 |  |  |  |  |  |
| 20* | 58 | M | PINS, L301 | Case | 2 | 70 | 150 | 2.5 | 18 | 9 | 50 | alleviation |  |
|  |  |  |  |  | 6 | 70 | 150 | 2.55 |  |  |  |  |  |
| 21 | 63 | M | PINS, L301 | Case | 4 | 60 | 130 | 3.1 | 24 | 14 | 41.67 | alleviation |  |
|  |  |  |  |  | 8 | 60 | 130 | 3.1 |  |  |  |  |  |
| 22 | 63 | M | PINS, L301 | Case | 2, 4 | 70 | 140 | 2.7 | 23 | 24 | -4.35 | deterioration |  |
|  |  |  |  |  | 6 | 70 | 140 | 2.9 |  |  |  |  |  |
| 23 | 72 | F | Medtronic, 3389S | Case | 2 | 70 | 120 | 2.15 | 16 | 11 | 31.25 | alleviation |  |
|  |  |  |  |  | 10 | 70 | 120 | 2.15 |  |  |  |  |  |
| 24 | 49 | F | PINS, L301 | Case | 3 | 80 | 130 | 2 | 24 | 0 | 100 | alleviation |  |
|  |  |  |  |  | 6 | 80 | 130 | 2.1 |  |  |  |  |  |
| 25 | 62 | M | Medtronic, 3389S | Case | 2 | 80 | 120 | 3.3 | 14 | 13 | 7.14 | minor change |  |
|  |  |  |  |  | 10 | 90 | 120 | 2.8 |  |  |  |  |  |
| 26 | 68 | F | Medtronic, 3389S | Case | 1 | 90 | 150 | 2.8 | 15 | 0 | 100 | alleviation |  |
|  |  |  |  |  | 9 | 90 | 150 | 2.8 |  |  |  |  |  |
| 27 | 69 | M | PINS, L301 | Case | 4 | 60 | 130 | 2 | 16 | 1 | 93.75 | alleviation |  |
|  |  |  |  |  | 6 | 60 | 130 | 2.35 |  |  |  |  |  |
| 28 | 68 | F | Medtronic, 3389S | Case | 2 | 70 | 145 | 2.5 | 20 | 22 | -10 | deterioration |  |
|  |  |  |  |  | 10 | 70 | 145 | 2.5 |  |  |  |  |  |
| 29 | 66 | F | PINS, L301 | Case | 2 | 60 | 155 | 2 | 23 | 22 | 4.35 | minor change |  |
|  |  |  |  |  | 7 | 90 | 155 | 3 |  |  |  |  |  |
| 30 | 67 | M | Medtronic, 3389S | Case | 2 | 60 | 110 | 2.8 | 11 | 5 | 54.55 | alleviation |  |
|  |  |  |  |  | 10 | 70 | 110 | 2.8 |  |  |  |  |  |
| 31 | 73 | M | PINS, L301 | Case | 4 | 100 | 160 | 3.5 | 23 | 0 | 100 | alleviation |  |
|  |  |  |  |  | 7 | 100 | 160 | 2.9 |  |  |  |  |  |
| 32 | 67 | M | PINS, L301 | Case | 1 | 70 | 90 | 3.75 | 24 | 24 | 0 | minor change |  |
|  |  |  |  |  | 6 | 70 | 90 | 3.75 |  |  |  |  |  |
| 33 | 72 | F | Medtronic, 3389S | Case | 1 | 90 | 145 | 3.05 | 10 | 8 | 20 | minor change |  |
|  |  |  |  |  | 9 | 80 | 145 | 1.8 |  |  |  |  |  |
| 34 | 58 | M | Medtronic, 3389S | Case | 2 | 70 | 145 | 2.3 | 14 | 13 | 7.14 | minor change |  |
|  |  |  |  |  | 11 | 70 | 145 | 2.3 |  |  |  |  |  |
| 35 | 64 | M | PINS, L301 | Case | 1 | 70 | 90 | 2.5 | 16 | 15 | 6.25 | minor change |  |
|  |  |  |  |  | 5 | 70 | 90 | 2.6 |  |  |  |  |  |
| 36 | 51 | M | Medtronic, 3389S | Case | 2 | 70 | 130 | 2.5 | 7 | 11 | -57.14 | deterioration |  |
|  |  |  |  |  | 10 | 60 | 130 | 1.9 |  |  |  |  |  |
| 37 | 63 | F | PINS, L301 | Case | 3 | 80 | 150 | 2.6 | 16 | 7 | 56.25 | alleviation |  |
|  |  |  |  |  | 7 | 70 | 150 | 2.4 |  |  |  |  |  |
| 38 | 57 | M | Medtronic, 3389S | Case | 3 | 70 | 90 | 2.3 | 17 | 12 | 29.41 | minor change |  |
|  |  |  |  |  | 11 | 70 | 90 | 2.3 |  |  |  |  |  |
| 39 | 64 | M | Medtronic, 3389S | Case | 2 | 60 | 135 | 2 | 17 | 6 | 64.71 | alleviation |  |
|  |  |  |  |  | 10 | 60 | 135 | 2.2 |  |  |  |  |  |
| 40 | 59 | M | Medtronic, 3389S | Case | 3 | 70 | 140 | 1.7 | 14 | 0 | 100 | alleviation |  |
|  |  |  |  |  | 9 | 70 | 140 | 2.3 |  |  |  |  |  |
| 41 | 63 | M | Medtronic, 3389S | Case | 3 | 70 | 135 | 2.3 | 19 | 13 | 31.58 | alleviation |  |
|  |  |  |  |  | 6 | 70 | 135 | 2.45 |  |  |  |  |  |
| 42 | 63 | M | PINS, L301 | Case | 2 | 20 | 157 | 3.05 | 7 | 12 | -71.43 | deterioration |  |
|  |  |  |  |  | 7, 8 | 100 | 157 | 4.35 |  |  |  |  |  |
| 43 | 62 | F | PINS, L301 | Case | 3 | 60 | 130 | 2 | 14 | 4 | 71.43 | alleviation |  |
|  |  |  |  |  | 7 | 60 | 130 | 2 |  |  |  |  |  |
| 44 | 66 | F | PINS, L301 | Case | 4 | 60 | 130 | 3 | 9 | 9 | 0 | minor change |  |
|  |  |  |  |  | 8 | 60 | 130 | 2 |  |  |  |  |  |
| 45 | 53 | M | PINS, L301 | Case | 2 | 70 | 145 | 2.1 | 22 | 12 | 45.45 | alleviation |  |
|  |  |  |  |  | 6 | 60 | 145 | 2 |  |  |  |  |  |
| 46 | 71 | M | PINS, L301 | Case | 3 | 70 | 120 | 2.2 | 22 | 7 | 68.18 | alleviation |  |
|  |  |  |  | 5 | 7 | 70 | 120 | 2.2 |  |  |  |  |  |
| 47 | 68 | F | PINS, L301 | Case | 2 | 80 | 90 | 2.6 | 22 | 19 | 13.64 | minor change |  |
|  |  |  |  |  | 5 | 80 | 90 | 2.6 |  |  |  |  |  |
| 48 | 50 | M | PINS, L301 | Case | 3 | 60 | 130 | 2.3 | 21 | 15 | 28.57 | minor change |  |
|  |  |  |  |  | 7 | 60 | 130 | 2.3 |  |  |  |  |  |
| 49 | 61 | F | PINS, L301 | Case | 4 | 60 | 130 | 2.5 | 24 | 20 | 16.67 | minor change |  |
|  |  |  |  |  | 8 | 70 | 130 | 3.2 |  |  |  |  |  |
| 50 | 68 | F | Medtronic, 3389S | Case | 3 | 60 | 125 | 3.3 | 24 | 2 | 91.67 | alleviation |  |
|  |  |  |  |  | 7 | 60 | 125 | 3.3 |  |  |  |  |  |
| 51 | 74 | F | PINS, L301 | Case | 4 | 60 | 90 | 1.5 | 22 | 22 | 0 | minor change |  |
|  |  |  |  |  | 8 | 60 | 90 | 2 |  |  |  |  |  |
| 52 | 69 | M | PINS, L301 | Case | 3 | 90 | 165 | 3 | 16 | 4 | 75 | alleviation |  |
|  |  |  |  |  | 8 | 60 | 130 | 3 |  |  |  |  |  |
| 53 | 68 | M | PINS, L301 | Case | 2 | 60 | 90 | 2.5 | 23 | 5 | 78.26 | alleviation |  |
|  |  |  |  |  | 6 | 90 | 90 | 2.3 |  |  |  |  |  |
| 54 | 57 | M | Medtronic, 3389S | Case | 4 | 90 | 155 | 3.3 | 16 | 1 | 93.75 | alleviation |  |
|  |  |  |  |  | 8 | 90 | 155 | 3.25 |  |  |  |  |  |
| 55 | 67 | F | PINS, L301 | Case | 2 | 60 | 90 | 1.8 | 24 | 13 | 45.83 | alleviation |  |
|  |  |  |  |  | 6 | 60 | 90 | 1.8 |  |  |  |  |  |
| 56 | 75 | M | Medtronic, 3389S | Case | 2 | 60 | 130 | 2.5 | 18 | 22 | -22.22 | deterioration |  |
|  |  |  |  |  | 5 | 60 | 130 | 2 |  |  |  |  |  |
| 57 | 69 | F | PINS, L301 | Case | 3 | 60 | 130 | 2 | 15 | 8 | 46.67 | alleviation |  |
|  |  |  |  |  | 6 | 60 | 130 | 2 |  |  |  |  |  |
| 58 | 64 | F | Medtronic, 3389S | Case | 3 | 70 | 130 | 3.3 | 15 | 2 | 86.67 | alleviation |  |
|  |  |  |  |  | 7 | 60 | 130 | 1.8 |  |  |  |  |  |
| 59 | 52 | F | PINS, L301 | Case | 2 | 60 | 135 | 2.55 | 8 | 3 | 62.5 | alleviation |  |
|  |  |  |  |  | 6 | 60 | 135 | 2.55 |  |  |  |  |  |
| 60 | 56 | M | Medtronic, 3389S | Case | 4 | 60 | 130 | 2.3 | 15 | 20 | -33.33 | deterioration |  |
|  |  |  |  |  | 8 | 60 | 130 | 1.4 |  |  |  |  |  |
| 61 | 67 | M | PINS, L301 | Case | 2 | 70 | 135 | 1.7 | 20 | 23 | -15 | deterioration |  |
|  |  |  |  |  | 6 | 70 | 135 | 1.7 |  |  |  |  |  |
| 62 | 71 | M | Medtronic, 3389S | Case | 4 | 70 | 145 | 2.65 | 9 | 0 | 100 | alleviation |  |
|  |  |  |  |  | 8 | 70 | 145 | 1.9 |  |  |  |  |  |
| 63 | 57 | F | PINS, L301 | Case | 3 | 60 | 135 | 1.65 | 10 | 7 | 30 | minor change |  |
|  |  |  |  |  | 8 | 60 | 135 | 2.2 |  |  |  |  |  |
| 64 | 59 | M | PINS, L301 | Case | 3 | 80 | 90 | 2 | 20 | 3 | 85 | alleviation |  |
|  |  |  |  |  | 7 | 70 | 90 | 2.2 |  |  |  |  |  |
| 65 | 37 | F | PINS, L301 | Case | 3 | 70 | 150 | 2.1 | 20 | 14 | 30 | minor change |  |
|  |  |  |  |  | 7 | 80 | 150 | 1.8 |  |  |  |  |  |
| 66 | 72 | M | PINS, L301 | Case | 3 | 60 | 145 | 2.3 | 7 | 10 | -42.86 | deterioration |  |
|  |  |  |  |  | 7 | 60 | 145 | 1.7 |  |  |  |  |  |
| 67 | 56 | M | PINS, L301 | Case | 2 | 60 | 130 | 1.65 | 18 | 22 | -22.22 | deterioration |  |
|  |  |  |  |  | 6 | 60 | 130 | 2 |  |  |  |  |  |
| 68 | 75 | F | PINS, L301 | Case | 3 | 60 | 135 | 2.3 | 12 | 2 | 83.33 | alleviation |  |
|  |  |  |  |  | 7 | 60 | 135 | 2.3 |  |  |  |  |  |
| 69 | 48 | M | PINS, L301 | Case | 3 | 60 | 125 | 1.9 | 6 | 2 | 66.67 | alleviation |  |
|  |  |  |  |  | 7 | 60 | 125 | 1.9 |  |  |  |  |  |
| 70 | 59 | F | Medtronic, 3389S | Case | 3 | 50 | 150 | 2 | 19 | 8 | 57.89 | alleviation |  |
|  |  |  |  |  | 7 | 60 | 150 | 1.65 |  |  |  |  |  |
| 71 | 58 | F | PINS, L301 | Case | 2 | 60 | 140 | 2.2 | 13 | 14 | -7.69 | deterioration |  |
|  |  |  |  |  | 6 | 60 | 140 | 2.55 |  |  |  |  |  |
| 72 | 56 | M | Medtronic, 3389S | Case | 3 | 80 | 105 | 2.9 | 8 | 7 | 12.5 | minor change |  |
|  |  |  |  |  | 7 | 80 | 105 | 2.3 |  |  |  |  |  |
| 73 | 57 | F | Medtronic, 3389S | Case | 3 | 70 | 130 | 3 | 11 | 12 | -9.09 | deterioration |  |
|  |  |  |  |  | 7 | 70 | 130 | 2.05 |  |  |  |  |  |
| 74 | 63 | F | PINS, L301 | Case | 3 | 70 | 165 | 2 | 21 | 2 | 90.48 | alleviation |  |
|  |  |  |  |  | 7 | 70 | 165 | 2.65 |  |  |  |  |  |
| 75 | 54 | M | PINS, L301 | Case | 2 | 60 | 132 | 1.3 | 19 | 18 | 5.26 | minor change |  |
|  |  |  |  |  | 6 | 60 | 132 | 2.2 |  |  |  |  |  |
| 76 | 65 | M | PINS, L301 | Case | 4 | 60 | 130 | 2.4 | 16 | 6 | 62.5 | alleviation |  |
|  |  |  |  |  | 6 | 60 | 130 | 2.1 |  |  |  |  |  |
| Overall | 62.40±8.66 | 37F/39M | 49P/27M | / | / | 67.63±11.30, 70.00±11.43 | 131.34±21.24, 130.88±19.86 | 2.36±0.56, 2.41±0.52 | 16.89±5.46 | 10.05±7.16 | 37.93±42.61 | 42/22/12 |  |

FOG-Q: Freezing of Gait Questionnaire; *Patient in constant current mode, the unit of amplitude is mA.

**Supplementary Table 2** Pre-operative characteristics and therapeutic stimulation settings in FOG Patients with Parkinson’s Disease

|  | FOG-alleviation patients | FOG-minor-change patients | FOG-deterioration patients | *p* value |
| --- | --- | --- | --- | --- |
| Gender (M/F) | 19M/23F | 12M/10F | 8M/4F | 0.398 |
| Age at surgery (yr) | 63.5 (10.5) * | 63 (11.5) | 63 (12) | 0.948 |
| Pre-op FOG-Q | 17.5 (7.75) | 18.5 (11) | 15.5 (8.5) | 0.277 |
| Pre-op MDS-UPDRS I | 16 (8.5) | 15.5 (11) | 20.5 (5.5) | 0.362 |
| Pre-op MDS-UPDRS II | 21 (9.75) | 20 (10) | 21.5 (8.25) | 0.582 |
| Pre-op MDS-UPDRS III (off medication) | 52 (38.2) | 48 (24) | 48 (18.8) |  |
| Pre-op MDS-UPDRS III (on medication) | 18 (22.8) | 23.5 (14.5) | 19.5 (11.8) | 0.675 |
| Pre-op MDS-UPDRS IV | 8.5 (13.5) | 9.5 (10.2) | 16 (13.8) | 0.488 |
| Pro-op MMSE | 26 (3) | 27 (3) | 26 (3.25) | 0.381 |
| Pre-op MoCA score | 23 (5) | 23.5 (4.75) | 22.5 (4.5) | 0.454 |
| HAM-D score | 17 (14) | 16 (9.75) | 22 (5.5) | 0.162 |
| HAM-A score | 18.5 (11.2) | 14.5 (11.8) | 19.5 (9.25) | 0.146 |
| Follow-up | 12 (15) | 12 (4.5) | 6 (6) | 0.092 |
| Right-side amplitude | 2.2 (0.637) | 2.42 (0.675) | 2.4 (0.462) | 0.763 |
| Right-side pulse width | 65 (10) | 70 (20) | 65 (10) | 0.398 |
| Right-side frequency | 132 (14.5) | 130 (55) | 132 (11.2) | 0.599 |
| Left-side amplitude | 2.3 (0.663) | 2.4 (0.75) | 2.02 (0.787) | 0.347 |
| Left-side pulse width | 70 (10) | 70 (20) | 65 (10) | 0.305 |
| Left-side frequency | 130 (12.2) | 130 (55) | 132 (11.2) | 0.607 |
| Post-op FOG-Q | 5 (6) | 14.5 (9) | 21 (10.2) | **< 0.001** |

*The data were shown as mean (SD).

**Supplementary Table 3** Coordinates of the active contacts in MNI space

|  | Right-side hemisphere | | | Left-side hemisphere | | |
| --- | --- | --- | --- | --- | --- | --- |
|  | X(mm) | Y(mm) | Z(mm) | X(mm) | Y(mm) | Z(mm) |
| 1 | 11.27 | -13.6 | -7.93 | -11.81 | -12.55 | -6.41 |
| 2 | 12.75 | -11.78 | -5.62 | -12.94 | -11.37 | -5.38 |
| 3 | 14.25//14.76 | -8.71//-7.53 | -6.36//-4.55 | -13.43//-13.81 | -12.30//-11.03 | -7.16//-5.44 |
| 4 | 13.17 | -10.78 | -3.19 | -14.21 | -14.47 | -3.95 |
| 5 | 13.38 | -10.42 | -3.77 | -12.62 | -9.31 | -5.56 |
| 6 | 11.91 | -11.64 | -4.51 | -12.76 | -12.97 | -4.21 |
| 7 | 14.55 | -14.44 | -4.5 | -13.24 | -12.24 | -5.78 |
| 8 | 11.79 | -13.84 | -8.14 | -13.41 | -12.65 | -7.61 |
| 9 | 14.47 | -12.67 | -2.78 | -13.93 | -11.2 | -3.36 |
| 10 | 12.73 | -11.29 | -6.19 | -13.19 | -10.14 | -5 |
| 11 | 12.22 | -14.31 | -4.49 | -14.77 | -12.33 | -4.61 |
| 12 | 10.93//12.00 | -15.45//-13.03 | -9.98//-6.21 | -11.52//-13.06 | -12.33//-10.23 | -8.10//-4.20 |
| 13 | 15.11 | -11.56 | -4.95 | -14.17 | -12.93 | -5.02 |
| 14 | 12.89 | -10.27 | -4.35 | -13.84 | -11.19 | -3.51 |
| 15 | 13.48 | -10.69 | -5.85 | -11.80//-12.34 | -14.06//-12.76 | -9.11//-7.29 |
| 16 | 12.31 | -11.67 | -4.94 | -12.35 | -10.93 | -4.15 |
| 17 | 12.3 | -12.14 | -4.42 | -12.82 | -13.22 | -5.13 |
| 18 | 13.89//14.45 | -12.71//-11.50 | -5.10//-3.14 | -13.61 | -11.21 | -4.45 |
| 19 | 11.75 | -13.41 | -5 | -12.2 | -13.81 | -6.58 |
| 20 | 12.97 | -14.37 | -5.54 | -11.42 | -14.48 | -6.71 |
| 21 | 12.88//13.41 | -12.77//-11.51 | -6.26//-4.34 | -12.86//-13.48 | -12.49//-11.28 | -6.65//-4.68 |
| 22 | 11.94//13.55 | -13.78//-11.42 | -7.78//-4.27 | -13.79 | -12.53 | -6.99 |
| 23 | 14.13 | -11.4 | -5.21 | -12.93 | -13.26 | -4.95 |
| 24 | 13.49 | -12.26 | -6.14 | -12.45 | -12.74 | -7.51 |
| 25 | 12.26 | -13.2 | -6.23 | -12.37 | -11.99 | -4.33 |
| 26 | 13.07 | -12.26 | -5.07 | -12.29 | -13.31 | -4.37 |
| 27 | 14.43 | -10.89 | -5.59 | -11.75 | -12.09 | -6.3 |
| 28 | 11.67 | -9.88 | -6.52 | -12.78 | -9.3 | -4.98 |
| 29 | 12.57 | -13.65 | -6.93 | -12.8 | -12.35 | -5.31 |
| 30 | 12.03 | -12.87 | -5.7 | -13.48 | -14.07 | -4.26 |
| 31 | 13.71 | -9.89 | -4.13 | -11.5 | -13.08 | -5.9 |
| 32 | 11.48 | -15.44 | -8.33 | -11.84 | -10.69 | -5.11 |
| 33 | 11.02 | -13.38 | -6.09 | -13.08 | -15.19 | -7.73 |
| 34 | 11.42 | -12.11 | -7.62 | -13.28 | -12.39 | -4.48 |
| 35 | 11.28 | -15.57 | -9.48 | -11.84 | -15 | -10.08 |
| 36 | 11.65 | -12.56 | -8.89 | -12.07 | -12.58 | -8.59 |
| 37 | 12.43 | -10.4 | -7.39 | -11.99 | -11.37 | -7.06 |
| 38 | 12.7 | -9.49 | -4.9 | -14.94 | -10.64 | -5 |
| 39 | 14.45 | -12.49 | -4.9 | -11.31 | -11.72 | -6.68 |
| 40 | 13.89 | -10.09 | -2.74 | -10.78 | -14.26 | -7.81 |
| 41 | 11.29 | -12 | -5.74 | -12.89 | -12.49 | -9.94 |
| 42 | 11.05 | -13.44 | -9.72 | -11.74//-11.81 | -7.37//-6.05 | -2.61//-0.56 |
| 43 | 13.96 | -12.53 | -6.3 | -11.29 | -12.53 | -7.28 |
| 44 | 12.96//15.23 | -14.42//-9.81 | -9.93//-4.00 | -12.21 | -11.03 | -5.26 |
| 45 | 10.57 | -15.47 | -10.66 | -12.23 | -13.5 | -8.68 |
| 46 | 13.21 | -13 | -7.58 | -12.01//-13.42 | -13.45//-11.30 | -10.30//-6.86 |
| 47 | 12.1 | -13.71 | -12.09 | -11.8 | -15.38 | -9.21 |
| 48 | 11.82 | -15.63 | -11.22 | -12.56 | -12.18 | -6.04 |
| 49 | 10.74 | -11.25 | -6.36 | -13.31 | -11.93 | -3.67 |
| 50 | 11.69 | -12.37 | -5.42 | -13.78 | -12.95 | -4.53 |
| 51 | 12.87 | -11.49 | -4.65 | -11.7 | -11.71 | -3.88 |
| 52 | 11.66 | -11.22 | -4.59 | -13.93 | -12.85 | -5.52 |
| 53 | 10.89 | -13.82 | -5.61 | -13.29 | -15.44 | -8.98 |
| 54 | 12.53 | -10.93 | -3.78 | -13.44 | -10.14 | -2.4 |
| 55 | 11.38 | -13.46 | -9.85 | -12.52 | -11.74 | -7.25 |
| 56 | 10.84 | -13.85 | -10.46 | -10.63 | -14.51 | -9.33 |
| 57 | 11.68 | -12.49 | -6.01 | -13.38 | -11.32 | -6.03 |
| 58 | 14.97 | -11.02 | -7.24 | -12.77 | -11.62 | -6.17 |
| 59 | 12.14 | -16.03 | -8.97 | -11.74 | -18.45 | -10.07 |
| 60 | 14.79 | -10.21 | -4.42 | -16.44 | -8.65 | -2.74 |
| 61 | 11.25 | -14.3 | -9.23 | -13.48 | -14.65 | -8.1 |
| 62 | 14.51 | -10.69 | -3.11 | -12.44 | -11.94 | -4.52 |
| 63 | 14.21 | -11.66 | -5.14 | -13.69 | -9.73 | -5.34 |
| 64 | 13.91 | -12.81 | -7.24 | -11.7 | -12.69 | -6.45 |
| 65 | 9.37 | -12.44 | -5.53 | -12.76 | -13.34 | -7.81 |
| 66 | 11.84 | -13.99 | -5.86 | -13.48 | -13.31 | -4.76 |
| 67 | 11.2 | -12.3 | -7.55 | -11.1 | -13.04 | -7.87 |
| 68 | 12.03 | -13.24 | -6.73 | -10.64 | -13.89 | -6.63 |
| 69 | 11.34 | -14.53 | -7.99 | -12.3 | -14 | -7.98 |
| 70 | 14.88 | -11.27 | -4.81 | -12.16 | -12 | -6.5 |
| 71 | 12.05 | -15.48 | -8.8 | -14.18 | -12.47 | -7.05 |
| 72 | 13.64 | -12.56 | -7.69 | -12.38 | -12.05 | -6.17 |
| 73 | 13.63 | -11.57 | -4.92 | -13.02 | -11.79 | -5.97 |
| 74 | 12.46 | -13.44 | -5.64 | -13.14 | -12.69 | -4.27 |
| 75 | 13.04 | -14.44 | -8.43 | -10.2 | -13.67 | -8.69 |
| 76 | 15.55 | -11.09 | -3.28 | -12.07 | -13.76 | -7.87 |
| Overall* | 12.66±1.31 | -12.47±1.63 | -6.27±2.05 | -12.7±1.06 | -12.45±1.71 | -6.11±1.86 |
| Alleviation | 12.96±1.31 | -12.25±1.57 | -5.81±1.72 | -12.59±0.88 | -12.68±1.47 | -6.24±1.69 |
| Minor change | 12.33±1.25 | -12.83±1.69 | -6.57±2.43 | -12.79±1.09 | -12.31±1.67 | -5.73±1.87 |
| Deterioration | 12.18±1.22 | -12.57±1.74 | -7.35±2.02 | -12.9±1.56 | -11.91±2.44 | -6.35±2.41 |

MNI: Montreal Neurological Institute; X coordinate: medial-lateral direction (lateral); Y coordinate: anterior-posterior direction (axial); Z coordinate: dorsal-ventral direction (vertical).

* Presented in Mean±SD.

**Supplementary Table 4** Correlations between the activated contacts’ coordinates and %FOG-Q change

|  |  | Regression with %FOG-Q change |  |
| --- | --- | --- | --- |
|  |  | *R* value | p Value |
|  | X | 0.27 | **0.020** |
| Right-side hemisphere | Y | 0.17 | 0.149 |
|  | Z | 0.32 | **0.005** |
|  | X | 0.05 | 0.650 |
| Left-side hemisphere | Y | 0.17 | 0.142 |
|  | Z | <0.05 | 0.979 |
